# The optical and biological properties of glacial meltwater in an Antarctic fjord

**DOI:** 10.1371/journal.pone.0211107

**Published:** 2019-02-06

**Authors:** B. Jack Pan, Maria Vernet, Rick A. Reynolds, B. Greg Mitchell

**Affiliations:** 1 Integrative Oceanography Division, Scripps Institution of Oceanography, University of California San Diego, La Jolla, California, United States of America; 2 Marine Physical Laboratory, Scripps Institution of Oceanography, University of California San Diego, La Jolla, California, United States of America; Shandong University, CHINA

## Abstract

As the Western Antarctic Peninsula (WAP) region responds to a warmer climate, the impacts of glacial meltwater on the Southern Ocean are expected to intensify. The Antarctic Peninsula fjord system offers an ideal system to understand meltwater’s properties, providing an extreme in the meltwater’s spatial gradient from the glacio-marine boundary to the WAP continental shelf. Glacial meltwater discharge in Arctic and Greenland fjords is typically characterized as relatively lower temperature, fresh and with high turbidity. During two cruises conducted in December 2015 and April 2016 in Andvord Bay, we found a water lens of low salinity and low temperature along the glacio-marine interface. Oxygen isotope ratios identified this water lens as a mixture of glacial ice and deep water in Gerlache Strait suggesting this is glacial meltwater. Conventional hydrographic measurements were combined with optical properties to effectively quantify its spatial extent. Fine suspended sediments associated with meltwater (nanoparticles of ~ 5nm) had a significant impact on the underwater light field and enabled the detection of meltwater characteristics and spatial distribution. In this study, we illustrate that glacial meltwater in Andvord Bay alters the inherent and apparent optical properties of the water column, and develop statistical models to predict the meltwater content from hydrographic and optical measurements. The predicted meltwater fraction is in good agreement with *in-situ* values. These models offer a potential for remote sensing and high-resolution detection of glacial meltwater in Antarctic waters. Furthermore, the possible influence of meltwater on phytoplankton abundance in the surface is highlighted; a significant correlation is found between meltwater fraction and chlorophyll concentration.

## Introduction

The physical impact of Antarctic glacial melting on sea level variability is being extensively studied [1] [2] [3] [4]. The effects of glacial meltwater on Antarctic coastal hydrography and regional marine ecosystem are also expected to be critical to future marine productivity, but understanding of these processes is limited. Meltwater is undoubtedly a significant feature as a consequence of atmospheric and oceanic warming in climate-sensitive polar regions [5]. In addition to sea level rise, the melting of both glacial and sea ice also induces water column stratification (particularly in shallow coastal regions), where the meltwater is likely to impact light availability, as well as the function and structure of food webs [6] [7] [8]. The Western Antarctic Peninsula (WAP) is a region experiencing rapid climate change [9]. Mean air temperatures along the WAP have increased significantly (1–2 °C) over the last 50 years [10], which has profound consequences on sea ice, ice shelves, and glacial melting [8] [11]. This change had already triggered the collapse of Larsen ice shelf A and B east of the Peninsula [12] [13] [14].

Fjord systems provide a unique opportunity for studying meltwater effect on coastal ecosystems. Fjords are submerged valleys established by erosion of glacial ice and sea level rise since the last glaciation, and they typically contain one or more sills and sediment-laden basins [15] [16]. Fjords with glaciers connected with the ocean, or tidewater glaciers, are common geographic features at mid- and high latitudes and they form an important glacio-marine boundary [17]. In polar regions, the physical transport in these semi-enclosed fjord systems serves as a major mechanism for moving glacial ice to the sea, hence they are sensitive to climatic warming and changes in ice-ocean dynamics [15] [18] [19]. These characteristics can result in a spatial gradient of meltwater from the glacial terminus to coastal ocean through the mouth of the fjord.

Initially, the transport and spatial distribution of meltwater in sub-Arctic fjords was considered to be consistent with a simple two-layer circulation–where warm oceanic water intrusion enters at depth, and then mixes with subglacial (i.e. at the glacier base) and submarine meltwater (i.e. generated at the glacier front) as it rises to the surface along the glacial terminus. The modified discharge water is transported out of the fjord and into the coastal ocean at the surface [20]. These processes broadly resemble those of an estuarine circulation. However, recent studies in Greenland fjords reveal that the physical oceanography and meltwater transport in glacial fjords are far more complex [21] [22]; for example, the subglacial discharge plume found in Saqqarliup Fjord (located near central western Greenland) is ~20m in diameter at surface of the glacial ice edge, but it spreads to a 200m by 300 m plume pool as it reaches the surface, before descending to its equilibrium depth [21].

Significant sediment plumes are associated with meltwater discharge; when the sediment plumes are prominent, they are detectable by ocean color sensors [23] and can be used as a proxy for estimating ice sheet runoff in Greenland [24]. Meltwater-induced turbid plumes can sometimes emerge at the surface near the glaciers [25] [26]. Suspended sediments from glacial meltwater ubiquitously contain iron (oxyhydr)oxide nanoparticles, typically ~5nm in diameter, and they can occur as single grains or aggregates that may be isolated or attached to sediment grains [27]. These discharged meltwater plumes not only have a significant impact on the coastal ocean in a fjord’s vicinity [28], but also on the global climate [25].

The meltwater plumes carrying inorganic particles, or “glacial flour”, have certain bio-optical characteristics. In western Greenland, Uummannaq Fjord and Vaigat–Disko Bay, freshwater influx from glacial terminus increases the diffuse attenuation coefficient of photosynthetically available radiation (K_d_(PAR)) and the optical backscattering coefficient within the water column [29]. The impact of a meltwater plume on underwater light field is so significant that PAR can be robustly modeled based on inorganic particle properties and chlorophyll-a (chl-a) concentrations alone [30]. Antarctic glacial meltwater can extend offshore >100m over the WAP shelf [8], but the plumes usually lack significant sediment load, due to lack of major sources of terrestrial runoff such as permafrost or riverine input, with the exception of penguin colonies [31]. In WAP fjords, including Andvord Bay, the cause of elevated beam attenuation coefficients along the glacial-marine interface have been identified as fine suspended sediment originating from the glaciers [17].

δ^18^O-salinity relationship has been used to study the evolution of water masses near ice shelves and to calculate the mean isotope ratio of melting ice by extrapolation of the mixing line to zero salinity [32] [33]. Once the meltwater has been identified, an effective method to track meltwater is by *in-situ* temperature and salinity distribution [34]. However, field methods are often limited in space and time. Therefore, it is important to develop additional means to monitor and track meltwater and assess its impact on regional scales. This beckons the utilization of non-conventional measurements to characterize meltwater and its spatial distribution. Optically derived relationships can allow *in-situ* data to be directly linked to remotely detectable variables, hence greatly expand the spatiotemporal range. Currently, the optical characteristics of Antarctic glacial meltwater are not well understood, in particular their inherent optical properties (IOPs) and how they are associated with other environmental variables. In this study, we aim to understand the spatial distribution of glacial meltwater throughout an Antarctic fjord, to characterize optical features associated with this meltwater in the water column, to quantify meltwater hydrography based on optical and physical measurements and finally, its impact on phytoplankton abundance.

## Material and methods

### Study area & field program

**Fig 1:**
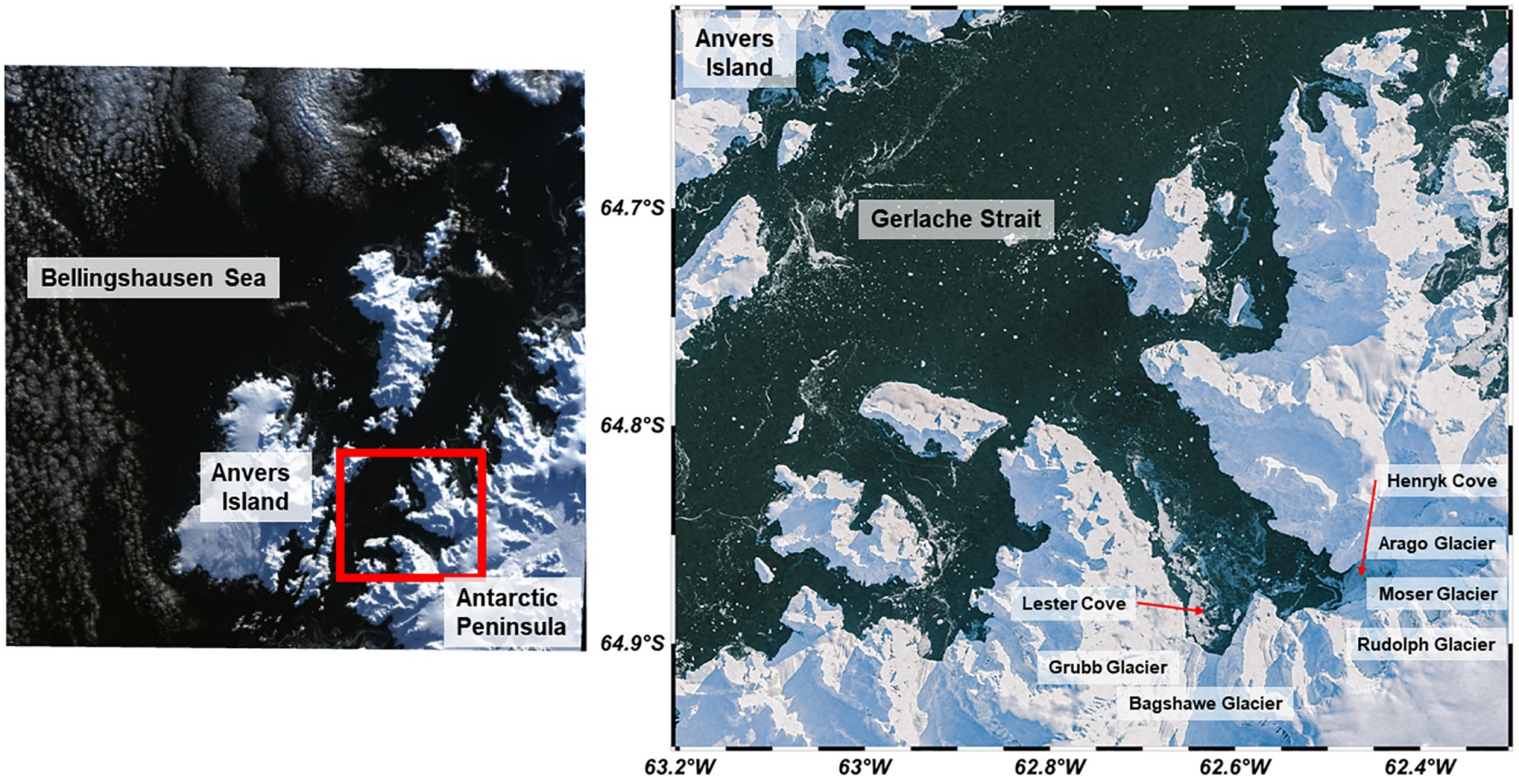
Map of the study region, Andvord Bay located on the Western Antarctic Peninsula. Map of Andvord Bay with colored contours depicting the fjord’s bathymetry. The distance from these glaciers in this study is calculated as the shortest displacement from a single glacial boundary line that encompasses all five glaciers in both Inner Basins. Satellite imagery retrieved from NASA/USGS Landsat 8 Scene ID LC82191052016109LGN00.

No specific permissions were required for these locations/activities presented in this manuscript. The field studies did not involve endangered or protected species. Sampling and data presented in this study were primarily collected in Andvord Bay, a fjord system in the WAP that is adjacent to Anvers Island and connected to Gerlache Strait (Fig 1). Andvord Bay is located on the WAP’s Danco Coast and is significantly glaciated, thus it is a distinct glacial ice drainage system [35]. The bay is historically free of sea ice during the summer months [36]. However, physical forcing, such as wind and currents, can temporarily cover the inner fjord with brash ice and icebergs. The geometry of Andvord Bay follows a typical fjord-type embayment. It is approximately 20 km in length, and has two inner basins (Inner Basin West at ~64.8918 ° S, 62.5973 ° W, and Inner Basin East at ~64.8731° S, 62.4476° W). There are several partial and full sills throughout this fjord which contribute to variability in its bathymetry. At the head of the fjord (at ~64.8959 ° S, 62.5397 ° W), there are five glaciers in direct contact with oceanic water; Grubb Glacier and Bagshawe Glacier drain into Inner Basin West, while Arago Glacier, Moser Glacier, and Rudolph Glaicer drain into Inner Basin East. These glaciers form an important interface where glacio-marine interactions occur. The distance from these glaciers is calculated as the shortest displacement from a single glacial boundary line that encompasses all five glaciers in both inner basins. Ice-modified water masses are in contact with Gerlache Strait water at the mouth of the fjord situated at ~64.7697 ° S, 62.7717 ° W, approximately 20 km away from the glaciers.

The field program in Andvord Bay was conducted onboard *R/V Gould* (LMG1510) and *RVIB Palmer* (NBP1603) within the FjordEco Program. Sampling on LMG1510 occurred between November 27^th^–December 20^th^, 2015, and then between April 4^th^—April 26^th^, 2016 on NBP1603, which coincided with late Austral spring and fall respectively. Daily station sampling and various meridional and zonal transects were conducted in Andvord Bay, Gerlache Strait, as well as at an open ocean station on the WAP shelf (Station B at ~ 64.7732 ° S, 65.3177 ° W, which overlaps with a shelf station on Line 600 in the Palmer LTER Program [37]). At each daily station and during transects, water samples and temperature-salinity-depth data were collected using a CTD rosette sampler with twenty-four 10-L bottles (Seabird SBE, Sea-Bird Electronics Inc., USA). Concurrently, light transmittance was measured by a CTD-mounted WebLabs C-Star transmissometer which was used to derive particulate beam attenuation coefficient (*c*_*p*_(660)); the transmissometer has a path length of 25 cm and operates at 660 nm. The CTD sampler was also equipped with a WetLabs FLRTD fluorometer to detect phytoplankton fluorescence and a Biospherical Instrument QSP-200L4S to measure photosynthetic active radiation (PAR) irradiance in the water column. Bio-optical samples were primarily collected within the euphotic layer, generally at depths of 50%, 10%, 1% of surface PAR irradiance; other water samples were collected at various 12 depths throughout the water column in order to opportunistically target certain hydrographic features of interest, as well as to follow pre-determined depths based on surface PAR attenuation.

Other instrument casts were conducted daily immediately before and after a CTD cast. A Profiling Reflectance Radiometer (PRR-800, Biospherical Inc., USA) and a Hydroscat-6 (HS-6, HOBI Labs, USA) were deployed to measure water column radiometric quantities and to estimate the spectral backscattering coefficient, *b*_*b*_(λ) (Table 1).

**Table 1:** Hydrological, biological, and bio-optical variables utilized in this study.

Variable	Definition	Unit
λ	Wavelength in vacuum	nm
	***Inherent Optical Properties (IOPs)***	
*a*_*p*_(λ)	Particulate absorption coefficient	m^-1^
*a*_*NAP*_(λ)	Detritus/Non-algal particulate absorption coefficient	m^-1^
*a*_*ph*_(λ)	Phytoplankton absorption coefficient	m^-1^
*a*_*CDOM*_(λ)	Colored dissolved organic matter absorption coefficient	m^-1^
*b*_*b*_(λ)	Backscattering coefficient	m^-1^
*b*_*b*,*sw*_(λ)	Seawater backscattering coefficient	m^-1^
*b*_*bp*_(λ)	Particulate backscattering coefficient	m^-1^
*c*_*p*_(660)	Particulate beam attenuation at 660nm	m^-1^
	***Apparent Optical Properties (AOPs)***	
*L*_*u*_(λ)	Upwelling Radiance	μW/(cm^2^ sr nm)
*E*_*d*_(λ)	Downwelling Planar Irradiance	μW/(cm^2^ nm)
*R*(λ)	Reflectance, ratio between *L*_*u*_(λ) and *E*_*d*_(λ) at discrete depths	sr^-1^
*R*_*rs*_(λ)	Remote sensing reflectance above surface	sr^-1^
K_d_(PAR)	Diffuse attenuation coefficient of PAR	m^-1^
	***In-situ Measurements***	
SPM_t_	Total suspended particulate mass concentration	mg/l
SPM_i_	Inorganic fraction of suspended particulate mass concentration	mg/l
SPM_o_	Organic fraction of suspended particulate mass concentration	mg/l
Chl-a	Chlorophyll-a pigment concentration	μg/l
Phaeo	Phaeo-pigment concentration	μg/l
δ ^18^O	Oxygen-18 isotope ratio	‰
Q_i_	Meltwater fraction	Decimal

### Radiometry and Apparent Optical Properties (AOPs)

PRR is a hand-deployed free-falling instrument that measures 16 spectral bands from 320nm– 670nm. It measures downwelling plane irradiance (*E*_*d*_*(λ)*, where λ is defined as light wavelength in vacuum), upwelling radiance from the nadir direction (*L*_*u*_*(λ)*), two PAR channels, as well as ancillary measurements such as depth, temperature and tilt/pitch angles of the instrument. A separate surface radiometer of this PRR package was setup on deck to record concurrent reference downwelling plane irradiance at surface (*E*_*s*_*(λ)*) during each deployment. Depending on weather and ice conditions, the instrument was deployed off the vessel’s stern, which was maneuvered to Sun-facing direction to avoid shadows of the research vessel. The instrument was allowed to drift more than 50m away from the ship before surface measurements were recorded, followed by the freefall cast to profile the water column. Each cast was performed by lowering the profiler until the maximum depth was obtained. One cast was recorded for each daily station.

Upwelling radiance reflectance normalized to downwelling irradiance (hereafter referred to as reflectance and symbolized as *R(λ)*) was calculated as the ratio between *L*_*u*_*(λ)* and *E*_*d*_*(λ)* at each respective wavelength and concurrent depths (z):

$$R(\lambda ,z)=\frac{L_{u}(\lambda ,z)}{E_{d}(\lambda ,z)}$$ (1)

where discrete *L*_*u*_*(λ*, *z)* is measured in unit of μW (sr cm^2^ nm)^-1^, *E*_*d*_*(λ*, *z)* is in unit of μW (cm^2^ nm)^-1^, and hence *R(λ*, *z)* has units of sr^-1^. Remote sensing reflectance (*R*_*rs*_*(λ)*) was calculated as:

$$R_{rs}(\lambda )=0.54\;\frac{L_{u}(\lambda ,0-)}{E_{s}(\lambda )}$$ (2)

where *L*_*u*_(*λ*, *0*−) indicates the value extrapolated to a depth just below the sea surface, and *E*_*s*_ is the above surface plane irradiance measured by the reference radiometer on deck. The advantage of this *R*_*rs*_*(λ)* calculation is that *E*_*s*_*(λ)* is not impacted by physical fluctuations which are common in underwater *E*_*d*_*(λ)* measurements (eg. wave focusing and vertical tilt); however, this method requires two instruments that are spatially separated, which may introduce bias due to moving clouds and ship’s shadows. Nevertheless, the radiometric sampling was conducted with a rigorous protocol and precise coordination with vessel operation to reduce these biases. This calculation is also consistent with the NASA protocol which has been implemented in the past; a detailed description of data processing has been described by Mitchell and Kahru [38].

### Bio-optical measurements and Inherent Optical Properties (IOPs)

#### Backscattering (*b*_*b*_(λ))

The spectral backscattering coefficient, *b*_*b*_*(λ)*, was obtained from the commercially available HS-6 developed by HOBI Labs [39]. HS-6 was winch-deployed on the starboard side of the research vessel. The instrument measures the volume scattering function (β) in six spectral bands (420, 442, 470, 510, 590, and 700 nm) at a scattering angle of 140°, which was then used to estimate *b*_*b*_*(λ)*. The backscattering coefficient is defined as the integral of β over the backwards hemisphere relative to the direction of light propagation [40],

$$b_{b}(\lambda )=2\pi \int_{\pi /2}^{\pi }\beta (\psi ,\lambda )\;sin(\psi )d\psi$$ (3)

where ψ is the scattering angle. The values of b_b_(λ) were estimated from the measurements of β(140°, λ) following the method by Doxaran *et al*. [41] and assuming a χ value of 1.13. Preliminary data processing was conducted in Hydrosoft 2.95. Because there is a finite distance between the instance of scattering and the detector, this measurement is also corrected for attenuation of the signal along the measurement’s path length, also known as “σ correction.” The σ correction procedure follows that described by Reynolds *et al*. [42].

To derive particulate backscattering coefficient (*b*_*bp*_*(λ)*), the effect of seawater backscattering needs to be excluded. Seawater backscattering (*b*_*b*,*sw*_*(λ)*) was calculated based on temperature and salinity measured by CTD at corresponding depths of each HS-6 cast [43]. *b*_*bp*_*(λ)* was then calculated as:

$$b_{bp}(\lambda )=b_{b}(\lambda )-b_{b,SW}(\lambda )$$ (4)

#### Absorption (*a*(λ))

Total particulate absorption (a_p_) was derived from laboratory measurements onboard each cruise. Optical density of filtered sample (OD_f_) was obtained by filtering water samples onto 25mm glass fiber filters (GF/Fs, ~0.8 μm pore size, Whatman) and scanning them in a spectrophotometer (UV/VIS PerkinElmer Lambda 18) from 200nm to 800nm with 1nm interval. An integrating sphere (Labsphere, Perkin−Elmer RSA−PE−18) was installed and each sample was measured in the transmittance port. Optical density of suspended sample (OD_s_) was derived and a_p_ was calculated from OD_s_ according to Stramski *et al*. [44] with moistened blank GF/Fs used as reference:

$$OD_{s}=0.679\;OD_{f}^{1.2804}$$ (5)

$$a_{p}=\text{ln}\left(10\right)OD_{S}\;\frac{A}{V}$$ (6)

where A is the sample-strained area on each GF/F, and V is the filtered volume of each water sample. The measured *a*_*p*_ was also partitioned into the absorption of non-algal particles (*a*_*NAP*_) and phytoplankton (*a*_*ph*_) through the use of methanol extraction according to Kishino *et al*. [45].

Absorption of CDOM (a_CDOM_) was measured by pressure-assisted filtration directly from the rosette bottles. Water samples were filtered through pre-combusted 47mm GF/Fs; each filter was heated to 450°C for 5 hours prior to the cruise. The sample filtrates were captured and stored in glass amber bottles that were cleaned with 10% HCl and then combusted before use. CDOM samples were measured in a 10cm cuvette by scanning in a dual-beam spectrophotometer (Cary 300 Agilent). Prior to each sample analysis, cuvette was rinsed with purified water (MilliQ) and then conditioned with sample water; MilliQ was used as the reference material. *a*_*CDOM*_ is derived from OD_s_ based on Mitchell *et al*. [46]:

$$a_{CDOM}=\frac{2.303\;OD_{S,CDOM}}{0.1}$$ (7)

### Chlorophyll-a

Phytoplankton abundance is estimated by the analysis of Chl-a concentrations. Water samples were filtered through Whatman GF/Fs under low vacuum, and immediately frozen at -80°C. This was followed by extraction of the pigments using 90% acetone solution, and measuring the fluorescence of each sample’s supernatant with a calibrated fluorometer (10AU Benchtop and Field Fluorometer, Turner Designs, USA). The calculation of Chl-a concentration from fluorescence was made according to Smith *et al*. [47].

### Suspended particulate mass

For suspended particulate mass concentrations (SPM), water samples were filtered under low vacuum through rinsed, combusted, and pre-weighed 25mm GF/Fs. Following filtration, filters and their edges were carefully rinsed with deionized water to remove residual sea salt and transferred to clean glass containers. Sample filters were dried at 60°C and stored in sealed containment until weighing. After determining SPM, the filters were combusted again in order to quantify the inorganic (SPM_i_) and organic (SPM_o_) fractions of each sample’s total SPM content.

### Meltwater fraction

Oxygen isotopic ratio (δ^18^O) samples were collected from discrete depths and are used to estimate the meteoric water fraction in Andvord Bay. Utilizing δ^18^O as a tracer to determine the source of water is based on the fractionation of the oxygen isotopes. Light water molecules, ^16^O, evaporate more readily than ^18^O, hence precipitation from this evaporation is enriched in ^16^O and depleted in ^18^O relative to the seawater standard. The δ^18^O of water samples are measured in the Stable Isotope Laboratory at Oregon State University. The sample is measured using the water-CO_2_ equilibration method modified from Epstein and Mayeda [48]. Standards are selected so that the range of expected sample δ^18^O values is bracketed. Sample vials are connected to the equilibration line and placed in a water bath (18°C), and allowed to temperature equilibrate for 15 minutes. After temperature equilibration, the headspace of the vials is pumped out for ~10 minutes, and then refilled with CO_2_ gas. Samples are then allowed to isotopically equilibrate for 12 hours. During the entire analysis, samples are slowly shaken to aid isotopic equilibration. The water samples are analyzed by dual inlet mass spectrometry using the DeltaPlus XL.

Results of discrete oxygen isotopic samples are reported in units of the standard per mil notation (‰) relative to the Vienna Standard Mean Ocean Water (VSMOW) [49]:

$$\delta^{18}O=(\frac{{\left(\frac{18_{O}}{16_{O}}\right)}_{sample}}{{\left(\frac{18_{O}}{16_{O}}\right)}_{standard}}-1)\times 1000‰$$ (8)

The analysis on the component sources of each water sample is based on the method from Jenkins and Jacobs [34, 50]. In this study, we are using δ^18^O as a one-dimensional domain to derive meteoric water fraction (Q_i_):

$$Q_{i}=\frac{\left(\delta^{18}O_{i}-\delta^{18}O_{GD}\right)}{\left(\delta^{18}O_{glacier}-\delta^{18}O_{GD}\right)}$$ (9)

where δ^18^O_i_ is the δ^18^O value of each discrete sample, δ^18^O_glacier_ is the δ^18^O value of one end-member obtained from glacial ice, and δ^18^O_GD_ is the averaged δ^18^O values from mid-depths sampled in Gerlache Strait as the other end-member (Table 2). If water column properties are a result of the mixing of glacial ice with an oceanic source, then the isotope composition of the mixture is given approximately by Eq 9. In general, it is assumed the mixing occurs between deep oceanic water of δ^18^O of ~0‰ with high salinity and a meteoric source of 0 PSU with low δ^18^O of (known as the Gade line, after Gade 1979 [51]). From Andvord Bay we measured glacial ice with δ^18^O of -12‰ in December and -15‰ in April (Table 2), similar to other WAP δ^18^O estimates of -16‰ [52] and -20‰ [53]. In order to increase the spatial resolution of meltwater fraction, each Q_i_ value was correlated with its corresponding salinity value in order to interpolate Q_i_ according to *in-situ* salinity for the entire Andvord Bay. In this study Q_i_ is reported in decimal fraction units instead of percentages.

**Table 2:** **Variables and their sample sizes (n) and standard errors (SE) utilized in the two-component mixing model for estimating meltwater fraction;** the two components are averaged values from glacial samples, and averaged mid-depth values from Gerlache Strait.

December 2016	δ^18^O (‰)	n	SE	Salinity (PSU)	n	SE
Gerlache	-0.17	7	0.01	34.508	7	0.013
Glacial melt	-12	5	1.16	0.000	--	--
**April 2017**						
Gerlache	-0.30	6	0.01	34.396	6	0.021
Glacial melt	-15	8	0.16	0.000	--	--

## Results

### Hydrography

**Fig 2:**
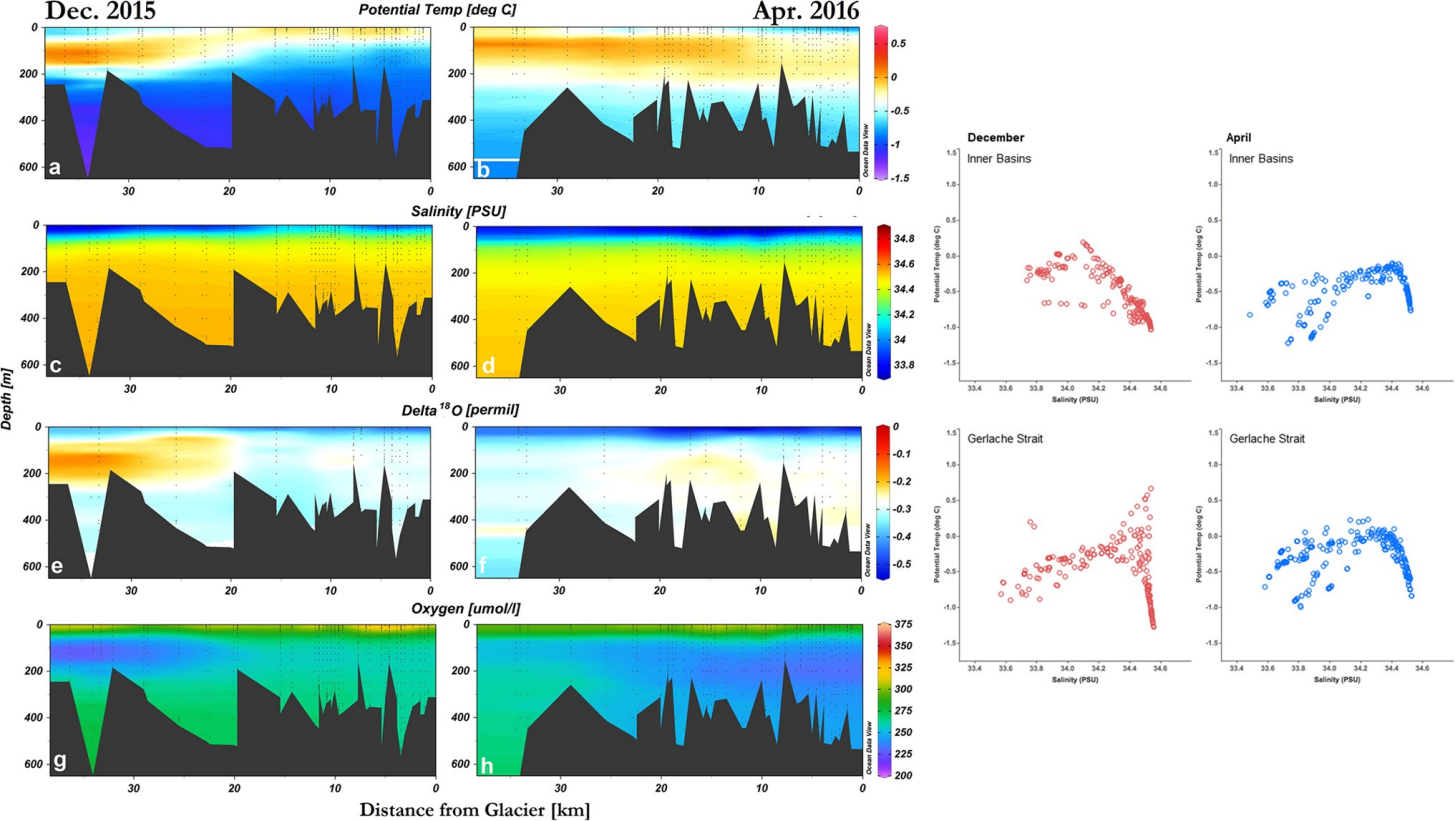
Overall hydrography profiles of Andvord Bay in December 2015, and April 2016. Left panels: Cross section plots of Andvord Bay depicting (**a, b**) potential temperature, (**c, d**) salinity, (**e, f**) oxygen isotope ratio, and (**g, h**) dissolved oxygen concentration, and as a function of their distance relative to the main glaciers situated in Andvord Bay. The differentiation between bottom topography is due to the slight differences in transect and sampling locations between the two study periods. Right panels: Temperature-salinity profiles of water masses within the inner basins and Gerlache Strait.

A cold surface layer, a relatively warmer sub-surface layer, and a cold deep water are present in Andvord Bay, and this feature is more prominent during April (Fig 2a and 2b). Overall, the average potential temperature of these 3 layers ≤ 20 km from the glaciers is -0.17 ± 0.01 °C (0–25 m), -0.54 ± 0.01 °C (25–200 m), -0.95 ± 0.004 °C (>200 m) for December and, -0.52 ± 0.01 °C (0–25 m), -0.17 ± 0.01 °C (25–200 m), -0.52 ± 0.01 °C (>200 m) for April, respectively, Within the Bay, the maximum and minimum potential temperature values are both observed in the Inner Basin West; the maximum and minimum of 0.19 °C and -1.22 °C were near the surface at 3.5–3.9 km from the termini. The average salinity of the surface, intermediate and deep layers was 34.05 ± 0.01 PSU (0–25 m), 34.41 ± 0.004 PSU (25–200 m), 34.53 ± 0.004 PSU (>200 m) for December, and 33.72 ± 0.007 PSU (0–25 m), 34.26 ± 0.007 PSU (25–200 m), 34.50 ± 0.007 PSU (>200 m) for April respectively, with a minimum of 33.47 PSU at 1.8 m depth, 12 km away from the glacial termini. The low salinity surface water is generally constrained to the fjord (Fig 2c and 2d). The surface layer also exhibits depletion in δ^18^O (z <25 m, -0.42 ± 0.009 ‰ for December and -0.51 ± 0.010 ‰ for April) while the rest of the water column presents relatively more enriched values (Fig 2e and 2f). The most depleted δ^18^O value of -0.66 ‰ was found at the surface (1.3 m depth) at mid-fjord (12 km away from the glaciers), and the most enriched value (-0.12 ‰) was found 33 km away. Higher dissolved oxygen concentrations are found at the surface, while relatively lower values and oxygen minimum are found at depth (Fig 2g and 2h). The maximum value of 370 μmol/L was observed at 2m depth in a middle basin. In summary, there is a warming and freshening with depleted δ^18^O from December to April, with the mean potential temperature increasing from -0.54 ± 0.01 °C to -0.36 ± 0.01 °C by April, the mean salinity decreasing from 34.34 ± 0.008 PSU to 34.11 ± 0.007 PSU (S1a and S1b Fig). The distinct surface fresh layer in December also deepens in April from 30 m to 60 m (Fig 2c and 2d). The mean δ^18^O value is -0.32 ± 0.006 ‰ in December and -0.36 ± 0.008 ‰ in April (S1c and S1d Fig).

### Phytoplankton

**Fig 3:**
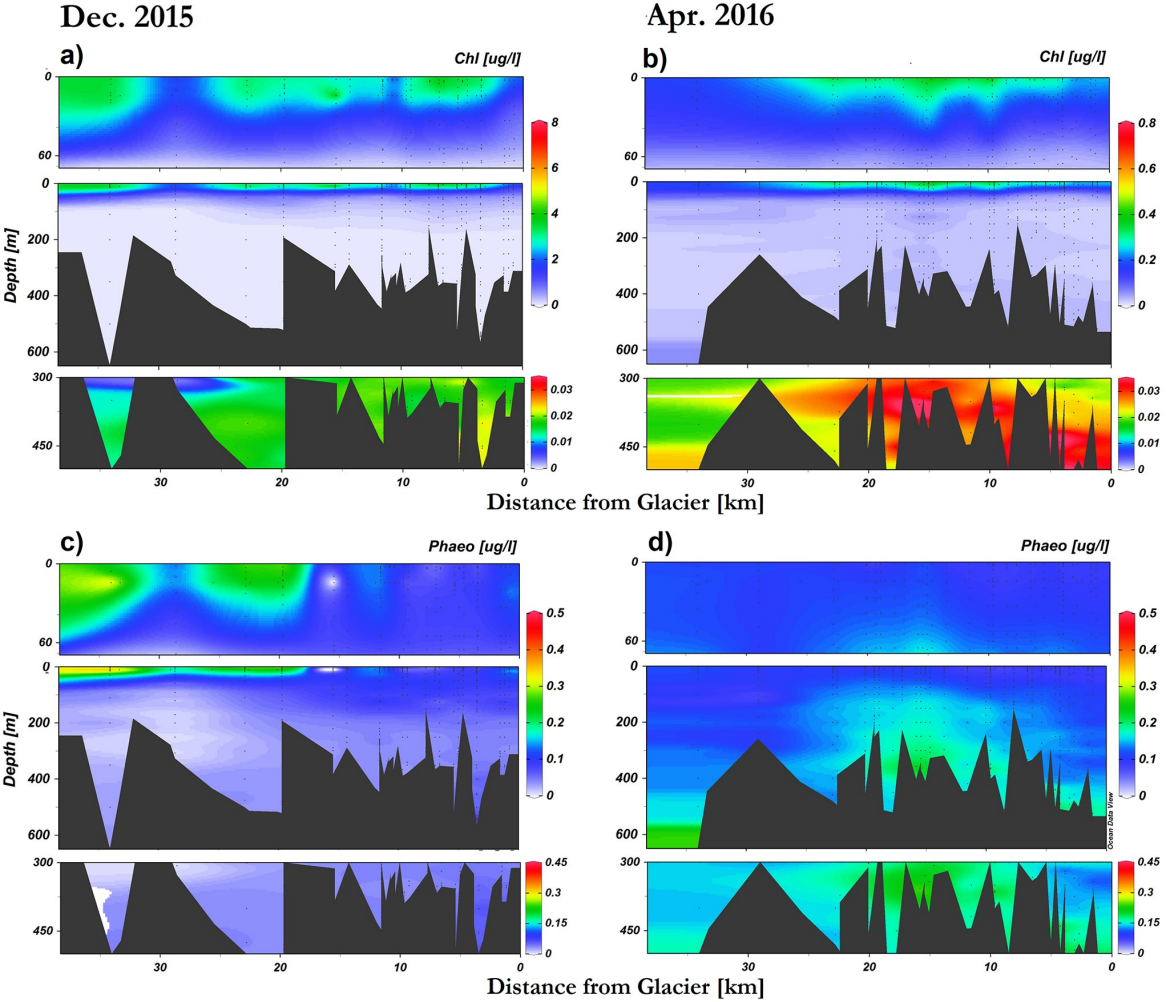
Chlorophyll-a concentration and phaeo-pigment concentration of Andvord Bay. (**a, b**) Chlorophyll-a concentration. (**c, d**) Phaeo-pigment concentration. Upper panel depicts surface profiles (0m – 100m), middle panel depicts the entire water column, and lower panel depicts deep profiles (300m – 500m). Depth axes in the surface plots are stretched to emphasize the euphotic layer. Appropriate scales are applied to certain color ramps to display data properly.

Highest chl-a concentrations occurred at the surface layer in Andvord Bay, mostly constrained to the fjord’s middle basins (Fig 3). Above 60 m depth, the average concentration was 1.211 ± 0.086 μg/l. The maximum value of 7.100 μg/L was found at 10 m depth and 12 km away from the glaciers coinciding with the minimum in salinity (Fig 2). Deep chl-a concentration (>300 m) followed a similar pattern, where high chl-a content was found in the middle and inner basins. Below 300 m depth, the overall concentrations varied from 0.002 μg/L to 0.061 μg/L. In contrast, surface maximum phaeo-pigment concentration occurred outside the fjord, in the Gerlache Strait (Fig 3). Above 60 m depth, the average phaeo-pigment concentration was 0.109 ± 0.003, ranging between 0.006 μg/L and 0.667 μg/L. The phaeo-pigment concentrations at depth (60 m to 300 m) generally occurred in the middle and inner basins. Below 300 m depth, the phaeo-pigment concentration varied from 0.005 μg/L to 0.265 μg/L.

There is a strong temporal variability pertaining to phytoplankton abundance in Andvord Bay. In December 2015, the mean chl-a concentration in the surface layer (0-60m) was 2.159 ± 0.144 μg/L (upper panel, Fig 3a), declining to 0.227 ± 0.009 μg/L in April (upper panel, Fig 3b). The overall mean chl-a concentration of the entire fjord was 1.063 μg/L in December decreasing to 0.122 μg/L in April (middle panels of Fig 3a and 3b). However, deep chl-a concentration (300 m—500 m) depicts a different temporal variability: there is an overall increase from 0.019 ± 0.001 μg/L in December to 0.029 ± 0.001 μg/L by April (lower panels of Fig 3a and 3b). In the case of phaeo-pigments, concentrations increased from December to April in the whole water column, from a mean of 0.087 ± 0.005 μg/L to 0.124 ± 0.002 μg/L by April. At depth (300 m– 500 m), mean phaeo-concentration increased 3-fold from 0.040 ± 0.002 μg/L (lower panel, Fig 3c) to 0.161 ± 0.006 μg/L (lower panel, Fig 3d).

### Optical properties of Andvord Bay

**Fig 4:**
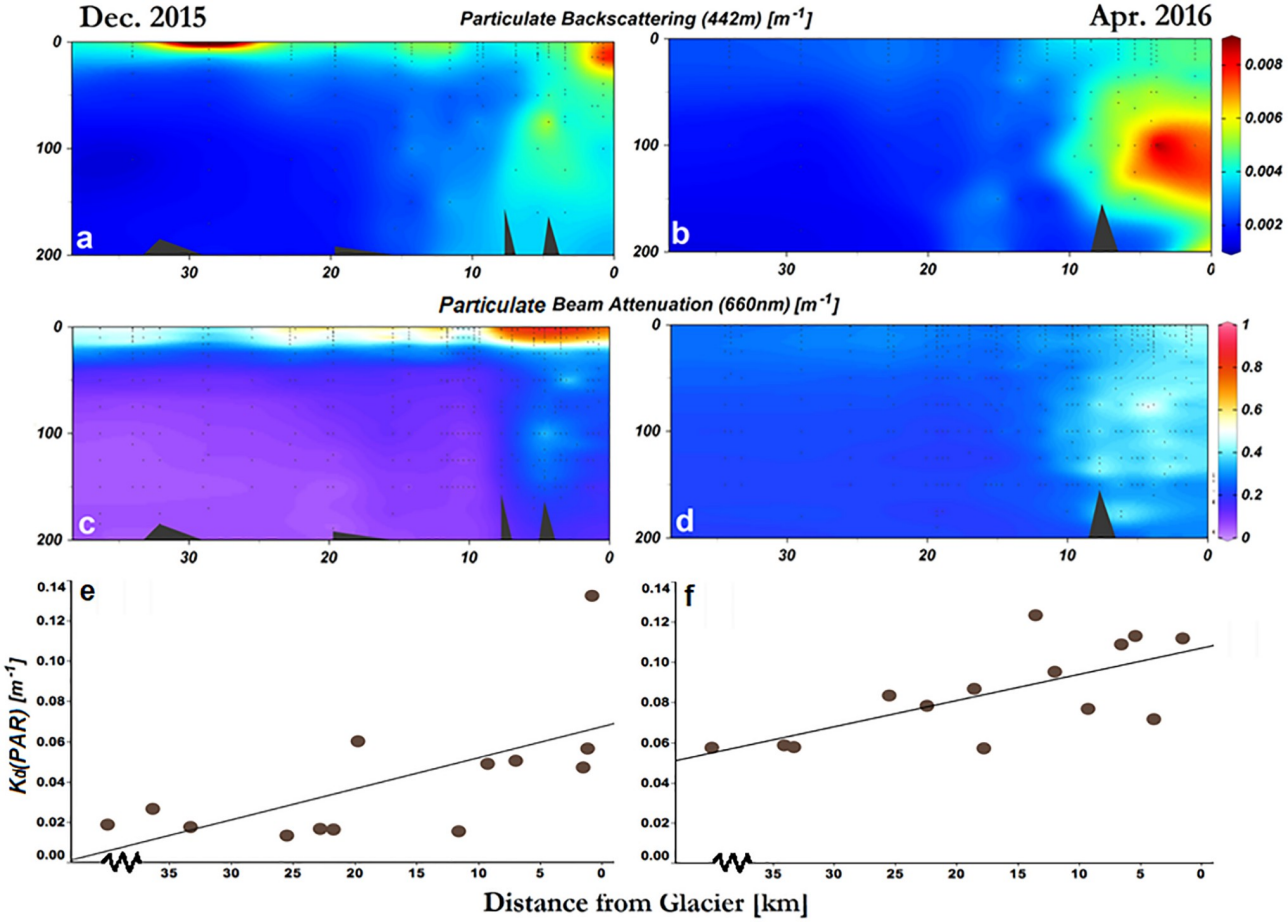
Spatial distribution of optical features in Andvord Bay. Profiles of particulate backscattering coefficient at 442nm and particulate beam attenuation coefficient at 660nm (**a–d**) in comparison to their corresponding diffuse attenuation coefficient of photosynthetically active radiation within the euphotic layer (**e, f**) in Andvord Bay during December 2015 and April 2016.

Distinct features are observed in profiles of particulate backscattering coefficient at 442nm (*b*_*bp*_(442)) as well as particulate beam attenuation coefficient at 660 nm (*c*_*p*_*(660)*); these features are primarily found at subsurface in the inner basins (Fig 4). For instance, the maximum b_bp_(442) value during the entire study period is 0.015 m^-1^, found in Inner Basin West at 100 m depth, 3.9 km away from the glacier termini (Fig 4b). Similarly, *c*_*p*_(660) presented a maximum of 2.213 m^-1^ in the Inner Basin West at 350 m depth and 3.09 km away from the glaciers (Fig 4c). On average, the b_bp_(442) and *c*_*p*_(660) decreased with distance from the glacier termini. In addition to the sub-surface features, there were also distinct surface optical signals (Fig 4a and 4b). Above 35 m depth, the maximum *b*_*bp*_(442) of 0.013 m^-1^ was found 0.8 km away from the glaciers, while most high *c*_*p*_(660) values aggregate at the surface and subsurface layers near the glacial front (Fig 4c and 4d). K_d_(PAR) was also higher near the glaciers (Fig 4e and 4f), however, its highest values coincided with an accumulation of chl-a near the fjord’s mouth (Fig 3b).

**Fig 5:**
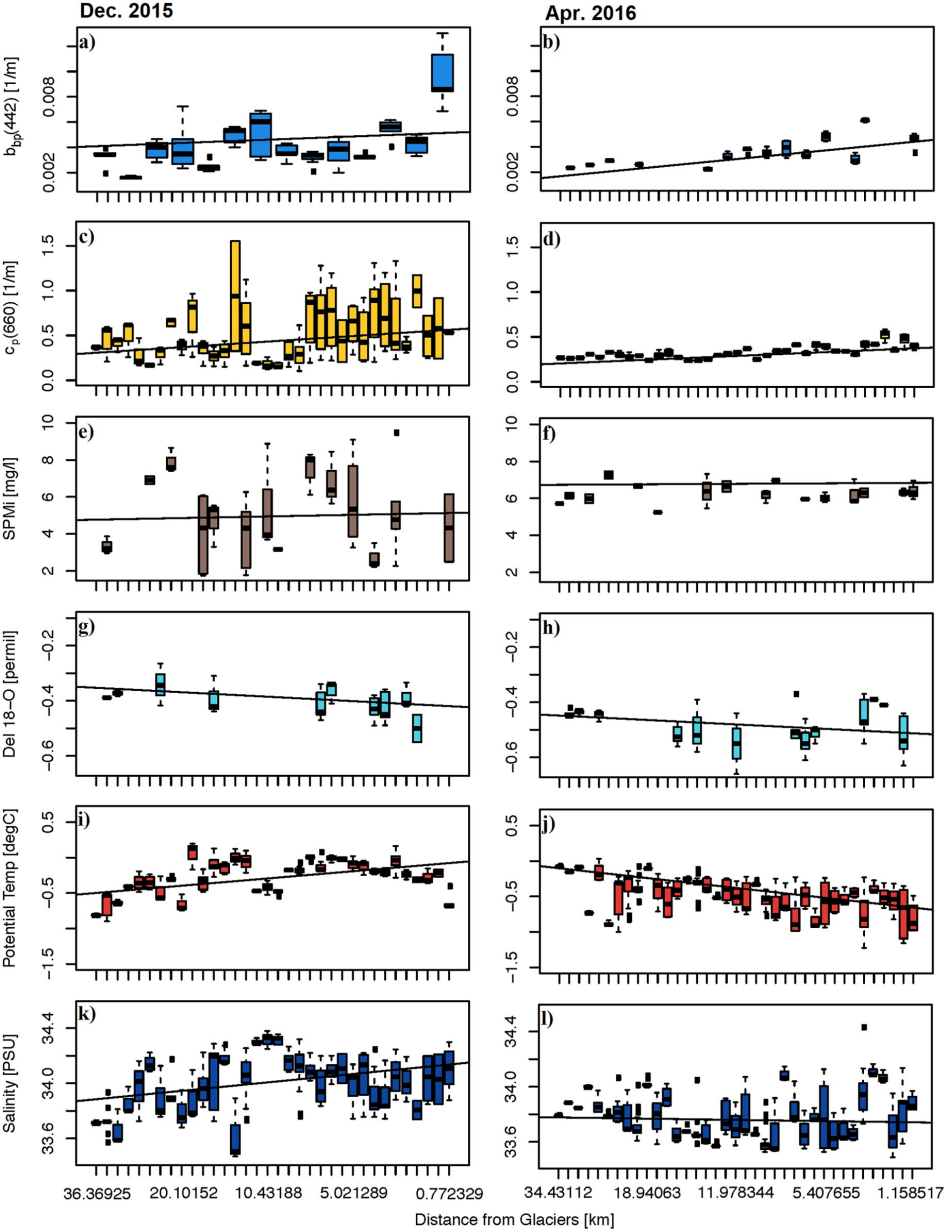
Profiles of optical and hydrographic variables. The box-whisker plots illustrate each variable’s range, variability at the sampling station, the overall trend towards the glaciers, and an overview of optical measurements and their coherence, or lack thereof, with physical variables. Each box represents a CTD profile along the fjord, and the bars on each box represent local maximum and minimum while the marker within each box represents local mean. Depth is between 0 m– 35 m and statistics pertaining to this figure are in Table 3.

In contrast to *b*_*bp*_(420) and *c*_*p*_(660) upward trend towards the glaciers (Fig 5a–5d, Table 3), inorganic suspended particulate mass concentration (SPM_i_) showed no statistically significant trend (Fig 5e and 5f; Table 3). However, δ^18^O was more negative towards the glaciers in both December and April (Fig 5g and 5h; Table 3). Potential temperature and salinity changed their trend towards the glacier front between December and April, from relatively warmer and higher salinity values near the termini (Fig 5i and 5k; Table 3) to fresher and colder (Fig 5j; Table 3).

**Table 3:** Statistical results derived from Fig 5, based on general linear regressions between physical/optical variables (at 0 m—35 m depth) and their corresponding locations relative to the glaciers at the head of Andvord Bay.

	December 2015			April 2016		
	r	slope	p-value	r	slope	p-value
*b*_*bp*_(420)	0.3797	-7.84E-05	<0.0001	0.6675	-7.58E-05	<0.0001
*c*_*p*_(660)	0.2007	-0.008	<0.001	0.5980	-0.005	<0.0001
δ^18^O	0.3579	0.027	<0.05	0.0927	0.002	<0.05
SPM_i_	0.0469	-0.011	0.7480	0.0141	-0.003	0.9388
Pot. Temp.	0.4982	-0.013	<0.0001	0.5144	0.015	<0.0001
Sal.	0.3351	-0.008	<0.0001	0.0510	0.001	0.3350
*R*(490)	0.3620	-0.0002	<0.01	0.5702	-0.0001	<0.0001
*K*_*d*_(PAR)	0.2420	-0.0057	<0.001	0.3149	-0.0025	<0.001

For each regression, correlation coefficient (r), the slope, and p-value are calculated to indicate the statistical significance of each slope, as well as to facilitate inter-comparisons among these variables.

When comparing phytoplankton abundance with optical properties, chl-a correlates with the downwelling diffuse attenuation coefficient at 443 nm (*K*_*d*_(443), p-value < 0.0001 for both December and April) but not with the backscattering coefficient, *b*_*bp*_(442) (p-value = 0.24 and p = 0.65, December and April respectively). However, chl-a is inversely correlated with water leaving radiance, or reflectance, *R*(443 nm, p-value < 0.001 for both cruises) while *b*_*bp*_(442) is positively correlated with *R*(443) (p-value < 0.001 in December and <0.0001 in April).

There is an overall higher particulate absorption coefficient at 442 nm (*a*_*p*_(442)) in December and April in the upper 100 m; the mean in December is 0.100 m^-1^ varying from 0.001 m^-1^ to 0.393 m^-1^. By April, mean *a*_*p*_(442) has decreased an order of magnitude to 0.015 m^-1^ (0.007 m^-1^–0.220 m^-1^) (S2a and S2b Fig). Non-algal particle absorption coefficient at 442nm (*a*_*NAP*_(442)) also exhibits an overall decline from 0.008 m^-1^ (0.001 m^-1^–0.023 m^-1^ in December) to 0.003 m^-1^ (0.001 m^-1^–0.006 m^-1^ during April) (S2c and S2d Fig). Conversely, CDOM absorption coefficient at 442nm (*a*_*CDOM*_(442)) increases from December to April (S2e and S2f Fig).

**Fig 6:**
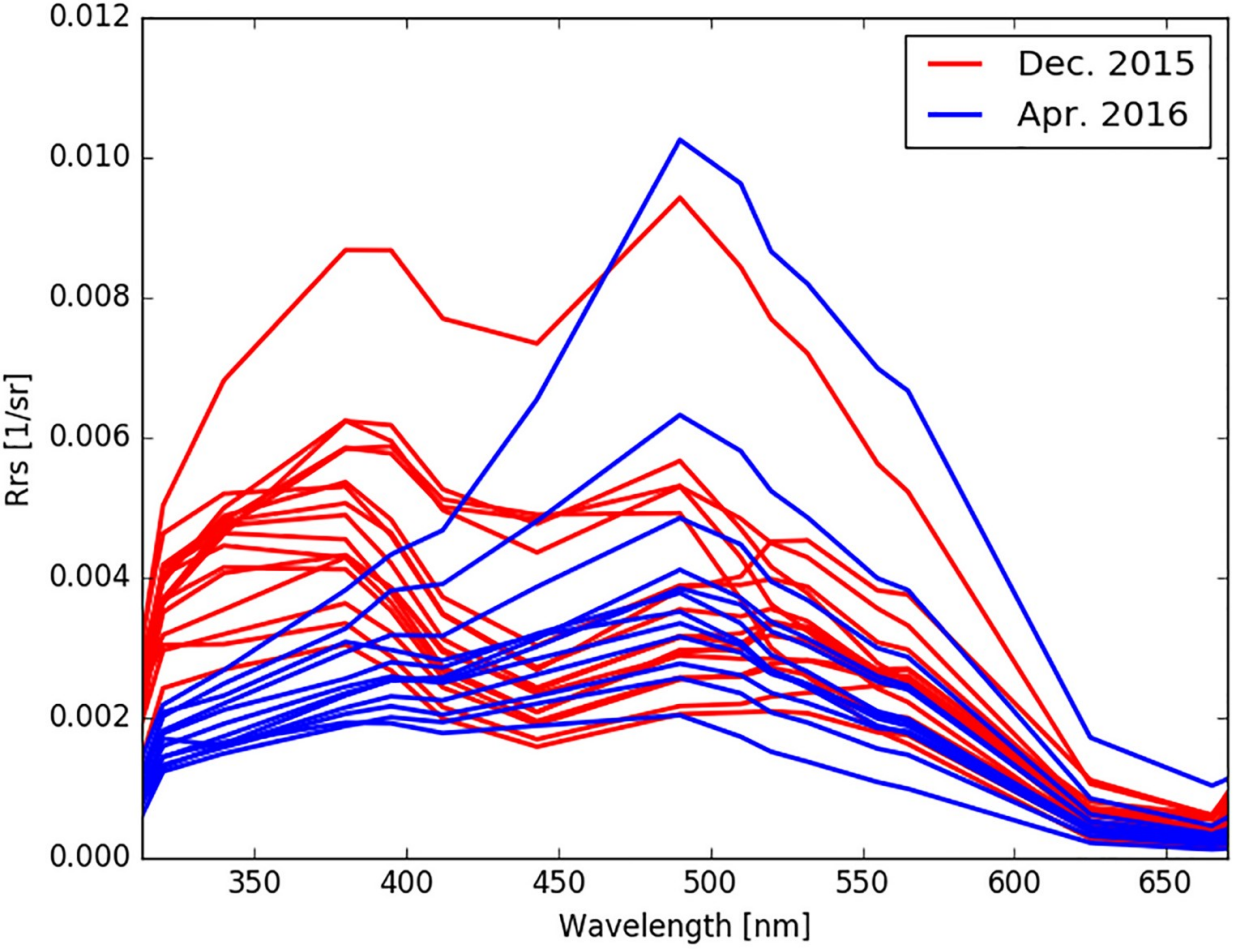
Remote sensing reflectance at the surface demonstrates a shift in spectral shape between December 2015 and April 2016.

In this way, a_*p*_(442) correlates with *c*_*p*_(660) during December (r = 0.93, p-value < 0.0001), while only *b*_*bp*_(442) correlates with *c*_*p*_(660) in April (r = 0.87, p-value < 0.0001). The change in particle assemblage from December to April is also illustrated in the spectral surface reflectance (*R*_*rs*_(λ)) (Fig 6). In December, a primary peak around 380 nm and a secondary peak around 490 nm were observed; these peaks are typical of certain phytoplankton pigments absorption in the blue spectral range [40]. However, the 380 nm peaks were absent in April and the primary peaks observed were at ~500 nm, indicating a coastal environment with sediment load [54].

### Meltwater fraction

Meltwater fraction for the entire bay was calculated based on the correlation between Q_i_ and salinity, First, meltwater fraction is calculated based on Jenkin’s method [34]; in this case, we utilized a two-component mixing model to estimate meltwater fraction based on δ^18^O (Eq 9). Then, in order to increase the spatial resolution of meltwater fraction, Q_i_, each value was correlated with its corresponding salinity value in order to generate a correlation to interpolate Q_i_ according to *in-situ* salinity. Due to a strong temporal difference between the two periods, two equations are derived for interpolating Q_i_ for December (Eq 10) and April (Eq 11) respectively.

$$Q_{i}=-0.016\;S+0.544$$ (10)

$$Q_{i}=-0.021\;S+0.740$$ (11)

Eq 10’s correlation coefficient is r = 0.82 with a p-value < 0.0001, while Eq 11’s is r = 0.95 with a p-value < 0.0001. Finally, based on these equations, Q_i_ data range is expanded by interpolation based on the salinity dataset.

**Fig 7:**
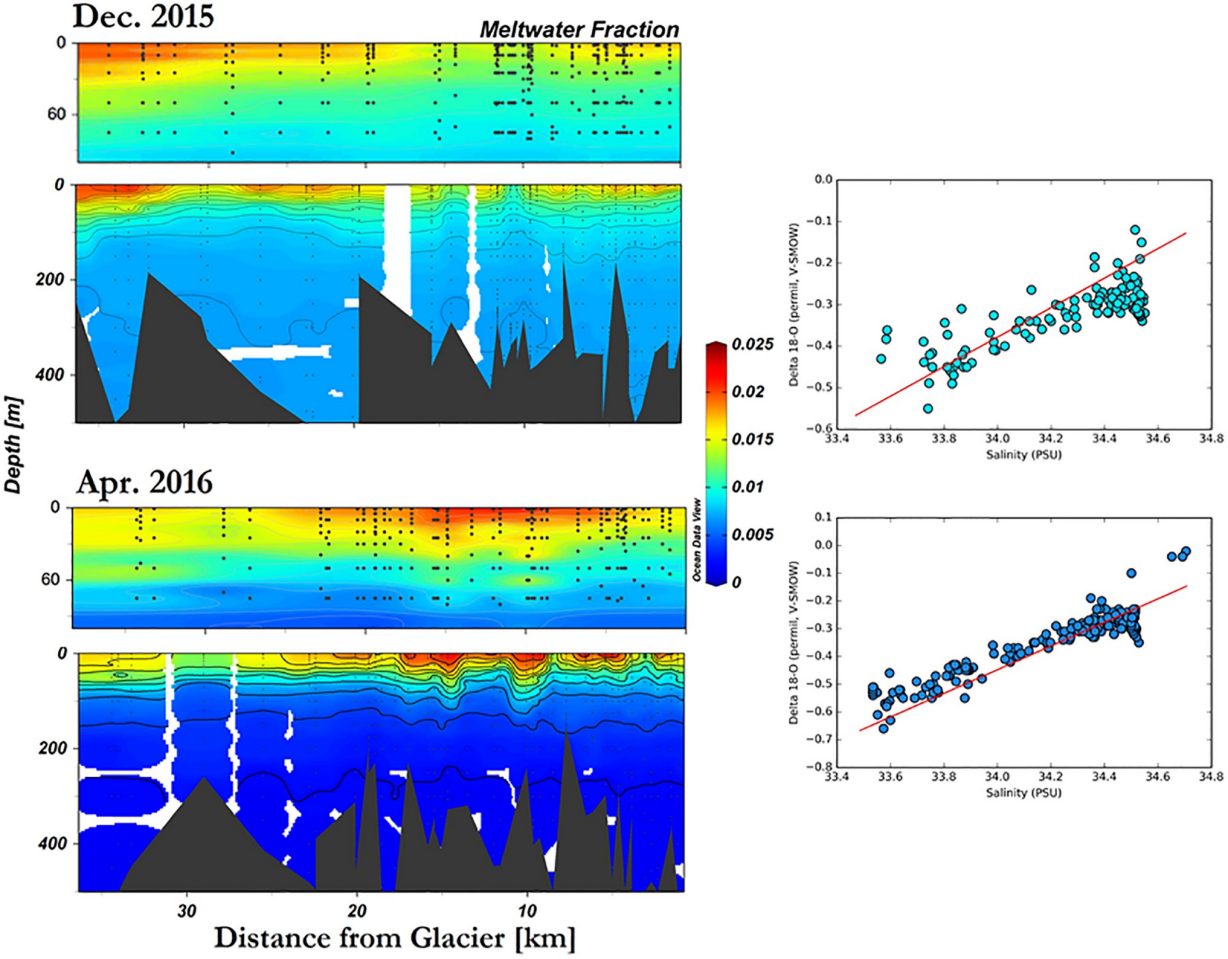
Meltwater fraction (Q_i_) of Andvord Bay in December 2015, and April 2016. Left panels: Q_i_ is calculated based on oxygen-18 isotope ratio data, and then interpolated according to salinity profile to extend data spatial resolution (upper panel: 0—100m, lower panel: entire water column). Right panels: Gade Line indicates two-component mixing. Detected by correlation between δ^18^O and salinity based on pure glacial meltwater and deep water of Gerlache Strait (Table 2); values sampled over the shelf are excluded in this calculation.

Meltwater fraction derived from δ^18^O values illustrates the fraction of glacial meltwater present in the water column of Andvord Bay (Figs 2 and 7). Overall, higher concentration of meltwater was found near the surface in the inner basins, within 100 m of the surface, with a mean of 0.012 ± 0.0001 in December and 0.015 ± 0.0003 in April. A maximum value of 0.024 was found at the surface in Inner Basin East during April, 2.3 km from the glacier. High meltwater was also found over the middle basins. Low meltwater fraction was typically found at depth. Tthe surface lens is persistent, increasing from December to April (Fig 7). Q_i_ integrated over 60 m within the fjord indicates that meltwater occupied 0.06 ± 0.003 m of the water column during December and 0.13 ± 0.017 m during April.

### Modeling of meltwater fraction

**Fig 8:**
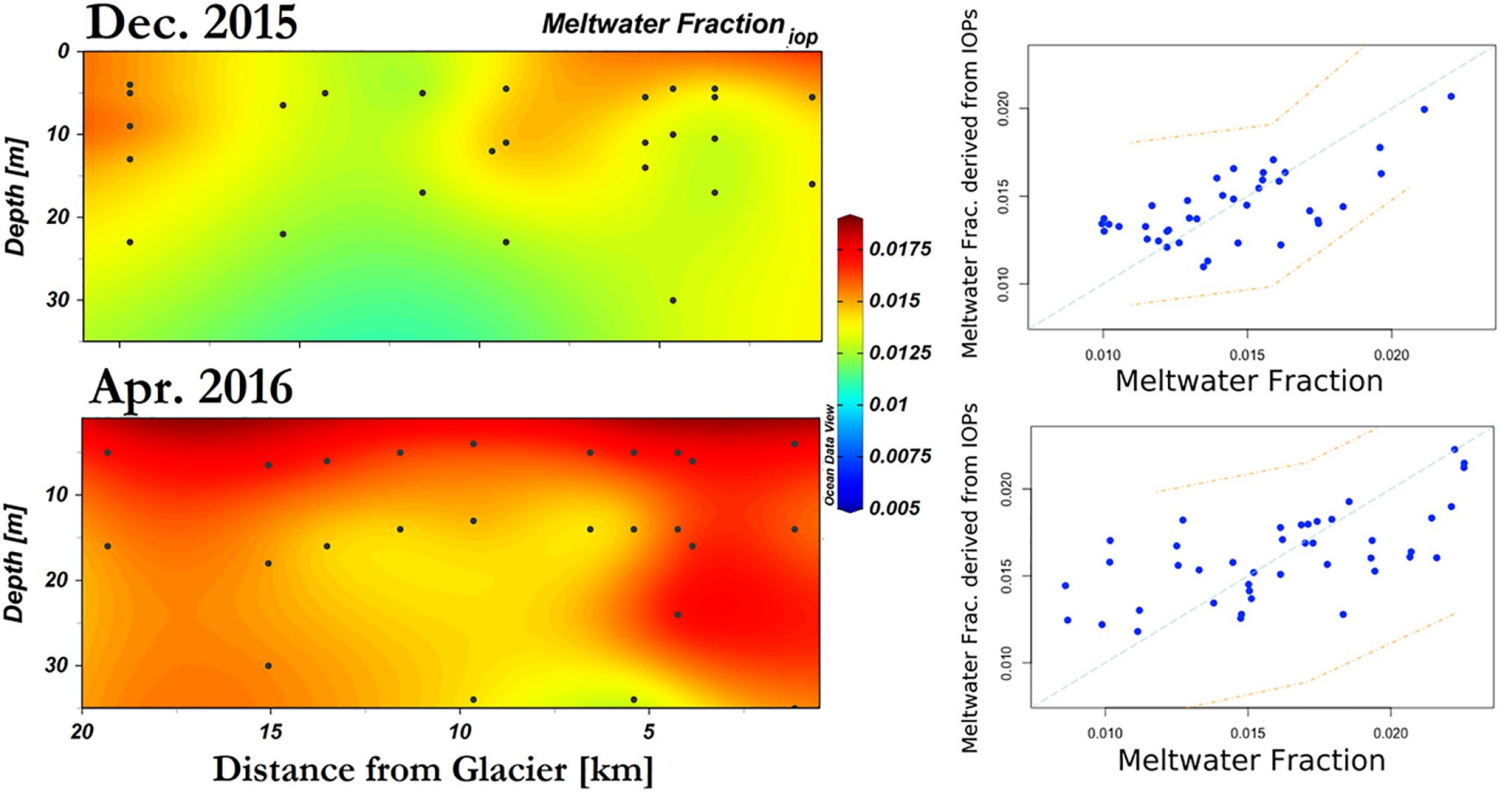
Section plots of meltwater fraction predicted based on Model 1 results. Left panels depict cross section plots of derived meltwater fraction from Model 1, which utilizes inherent optical properties–particulate backscattering coefficient at 442nm, non-algal particle absorption coefficient at 442nm, as well as temperature. Right panels depict their correlation with measured meltwater fraction based on oxygen-18 isotope ratios; orange dash lines indicate prediction intervals at 95% confidence.

A multiple-regression models was created in RStudio for predicting meltwater hydrographic features based on their IOPs. The IOP model (Model 1) is set as:

$$Q_{i,iop}=\beta_{1}+\alpha_{1}\;b_{bp}(442)+\alpha_{2}\;a_{NAP}(442)+\alpha_{3}\;Temp$$ (12)

where Q_i,iop_ is the predicted meltwater fraction, *b*_*bp*_(442) and *a*_*NAP*_(442) are utilized to encompass both backscattering and absorption of inorganic sediment particles known to be associated with glacial meltwater. Temp is in-situ temperature at the same depths (Fig 8). Various skill metrics were used to quantify model-data fits. The Pearson correlation coefficient (r) gives a measure of linear correlation between the measured and predicated variables. The root-mean-square deviation (RMSD) quantifies the scale of the difference between the model and *in-situ* data for any given point. Standard errors (SE) were also used as a measure of the statistical accuracy of the estimate. Prediction interval estimates a range in which a future observation will fall, with 95% certainty. For December, the model has a p-value of 3.52 E-05 with a RMSD of 0.002 (Table 4). In comparison, the April model has a p-value of 1.62 E-04 and a RSMD of 0.003. Model 1 predicts Q_i_ in December with an adjusted correlation coefficient of 0.66 and 0.60 in April (Table 4).

**Table 4:** Statistical results of predicted meltwater fraction when compared to *in-situ* observations. Derived Q_i,IOP_ from Model 1 is based on inherent optical properties, backscattering and non-algal particle absorption coefficients at 442nm, and temperature; while derived Q_i,AOP_ from Model 2 is based on reflectance band addition of 555 nm and 625 nm, and band ratio between 625 nm and 490 nm (a variation based on Sravanthi *et al*. [55]).

	December 2015	April 2016
**Model 1: Derived Q**_**i**_ **from IOPs**		
**Q**_**i,iop**_ **= β**_**1**_ **+ α**_**1**_ ***b***_***bp***_**(442) + α**_**2**_ ***a***_***NAP***_**(442) + α**_**3**_ **Temp**		
p-value	3.52E-05	1.62E-04
RMSD	0.002	0.003
Multiple r	0.698	0.638
Adjusted r	0.661	0.600
β_1_	0.012	0.012
α_1_	-0.310	-1.054
α_2_	0.267	1.530
α_3_	-0.006	-0.005
β_1_ SE	0.001	0.002
α_1_ *b*_*bp*_(442) SE	0.171	0.520
α_2_ *a*_*NAP*_(442) SE	0.071	0.373
α_3_ Temp SE	0.001	0.002
**Model 2: Derived Q**_**i**_ **from AOPs**		
${Q_{i,aop}=\beta_{2}+\alpha_{4}(R(555)+R(625))+\alpha_{5}(\frac{R(625)}{R(490)})}^{2}$		
p-value	< 2.2E-16	<2.2E-16
RMSD	0.002	0.003
Multiple r	0.821	0.849
Adjusted r	0.817	0.844
β_2_	1.428e-02	1.774e-02
α_4_	-3.077e-02	-6.599e-02
α_5_	7.532e-06	2.245e-06
β_2_ SE	2.168e-04	4.348e-04
*α*_4_(*R*(555) + *R*(625))SE	3.045e-03	6.915e-03
${\alpha_{5}(\frac{R(625)}{R(490)})}^{2}SE$	3.014e-06	1.929e-06

Model 2 is based on AOPs and it is a modified algorithm from Sravanthi *et al*. [55] for retrieving suspended sediment concentration in the Indian coastal ocean from remote sensing reflectance. Sravanthi *et al*.’s algorithm is based on a global algorithm originally published by Tassan [56]:

$$X_{s}=[R(\lambda_{i})+R(\lambda_{j})]{\left[\frac{R(\lambda_{m})}{R({\lambda_{}}_{n})}\right]}^{b}$$ (13)

where X_s_ is the suspended sediment concentration, *R*(λ) is remote sensing reflectance. λ’s are defined as spectral regions (rather than particular wavelengths) for the general purpose of future algorithm development. *λ*_*i*_ and *λ*_*j*_ are zones of low phytoplankton and CDOM absorption, respectively. *λ*_*m*_ and *λ*_*n*_ are choosen in the slope zone of phytoplankton absorption spectrum (where the slope is the steepest). However, Sravanthi *et al*. found that this global algorithm cannot produce results with high statistical significance, so they modified it to fit regional conditions:

$$Y=\beta +\alpha ([R(555)+R(620)]+{\left[\frac{R(620)}{R(490)}\right]}^{2})$$ (14)

Where Y is suspended sediment concentration, *λ*_*i*_ and *λ*_*j*_ from Eq 14 are 555nm and 620nm; these wavelengths are chosen because they represent low phytoplankton and CDOM absorptions in the Indian Ocean. *λ*_*m*_ and *λ*_*n*_ are 620nm and 490nm, to normalize any effect of phytoplankton particles on the overall reflectance signal.

In this study, further modifications were made on *λ*_*j*_ and λ_m_ in Eq 14 due the in-situ radiometer’s optical band configuration which only provided L_u_ and E_d_ at 625nm (used in the R(625) calculation, see Materials and methods section b). The final model is:

$${Q_{i,aop}=\beta_{2}+\alpha_{4}(R(555)+R(625))+\alpha_{5}(\frac{R(625)}{R(490)})}^{2}$$ (15)

Model 2 (Eq 15) predicts meltwater fraction based on a multivariate linear correlation, where Q_i,aop_ is the predicted meltwater fraction based on AOPs, and β_2_ is the intercept while α_4_ and α_5_ are the slopes for band addition and ratio respectively. Model 2 for April has a p-value of < 2.2 E-16 and RMSD of 0.003, predicting Q_i_ values, with an adjusted correlation coefficient of 0.844 while the December model has a correlation coefficient at 0.817 and a p-value < 2.2E-16 and RMDS of 0.002 (Table 4).

**Fig 9:**
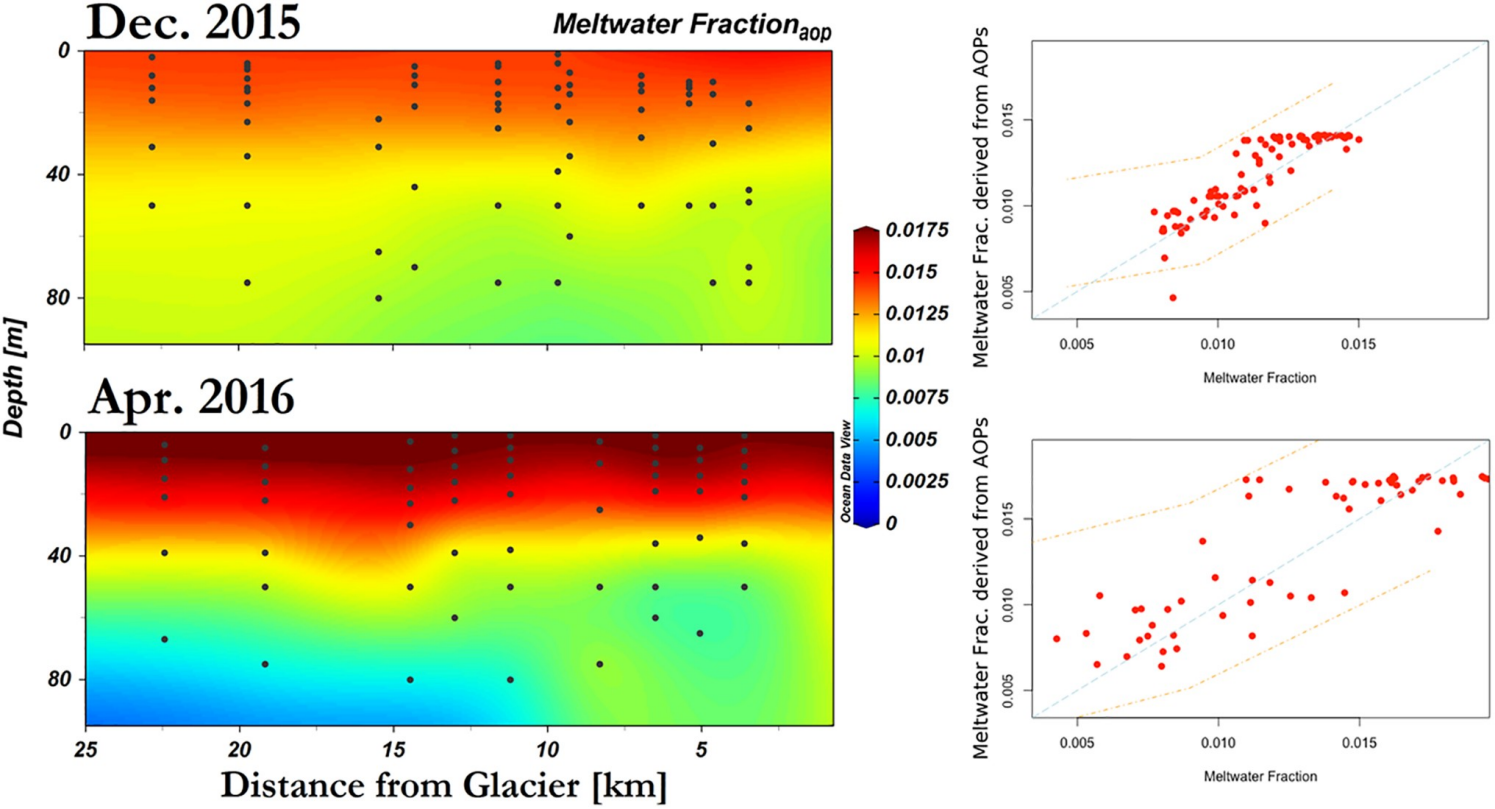
Section plots of meltwater fraction predicted based on Model 2 results. Left panels depict cross section plots of derived meltwater fraction from Model 1, which utilizes reflectance signal band ratio and addition. Right panels depict their correlation with measured meltwater fraction based on oxygen-18 isotope ratios; orange dash lines indicate predication intervals at 95% confidence.

In summary, these two models, based on physical and optical parameters, are able to reproduce the variability in meltwater fraction from December to April as well as its spatial variability away from the glacier front (Figs 8 and 9).

## Discussion

### Meltwater properties

The low salinity surface layer in Andvord Bay, persistent from December to April, in combination with lower δ^18^O and higher *b*_*bp*_(442) suggests this surface lens might have a glacial origin. Its specific origin is uncertain, it could originate from surface glacial or subglacial meltwater, that is from glacier processes, and/or from melting of brash ice and icebergs in the bay which originate from glacier calving. The fresher and cooler surface lens has been identified in the WAP as “meteoric” water, based only on salinity and δ^18^O properties [57, 58]. The optical properties of this freshwater lens, included in this study, and the proximity of this lens to the Bagshawe glacier provide additional support to identify it as glacial meltwater, with the caveat that precipitation, mainly as snow, directly over the ocean or as input from the fjord’s edges, could have contributed to the meltwater signal.

Meltwater fraction calculation was based on a two-component mixing model by Jenkins [1999] (Table 2). This model assumes all water within the fjord is a result of mixing between these two sources [34]. The meltwater fraction derived from δ^18^O samples is then linearly related to salinity profiles; this is based on the “Gade Line” method which assumes that salinity is largely influenced by two defined sources [51]. A closer examination of the correlation between δ^18^O and salinity reveals that the two properties are significantly more correlated in April 2016 than in December 2015, which are likely due to melting during the austral summer. The general linearity of all data points along the Gade Line for April (Fig 7) indicates a two end-member mixing with little influence from a third source (i.e. slight deviation in Gade Line during December). Moreover, temperature-salinity diagrams of Andvord Bay indicate the water mass found in the inner basins are different from that found in Gerlache Strait during December (Fig 2, right panels). However, the water masses in the two locations become more similar in April; this change in T-S and the similarity between the two locations further illustrate the mixing of glacial meltwater during summer. Mortensen *et al*. has observed a water mass’ T-S signal in Kangersuneq, SW Greenland during summer that is similar to Andvord Bay in April [57].

Meltwater in Andvord Bay shares similar physical characteristics with properties found in other fjords, mainly those in the Arctic and Greenland; however, the magnitude of these characteristics as well as their impact on the water column optics and the spatial distribution of phytoplankton abundance differ significantly. Pure meltwater of glacier origin is known to have characteristics of colder temperature, lower salinity, and higher turbidity in comparison to ambient oceanic water masses [8] [25] [57]. In contrast, upwelling processes at the glacier front could bring up to the surface warmer, more saline waters, depending on the properties of the deep water entering the fjord [22]. In Andvord Bay, we observed a consistent freshening at the surface layer (Fig 2c and 2d), associated with a decrease in temperature. However, the gradient of salinity along the fjord is not prominent (Fig 5l) in comparison to fjords with significant freshwater influx [25]. In Andvord Bay, there is an estimated 2–4 x10^6^ m^3^/day of solid ice flux from Bagshawe Glacier into Inner Basin West, but a low meltwater flux is expected (pers. comm. Truffer, M., University of Alaska, Fairbanks). The meltwater fraction in Andvord Bay (an average of 1.85% ± 0.29% in April) also suggests a weaker melting process than that observed in western Greenland fjords, in line with Marguerite Bay estimates where the percentage of meteoric water at 15m depth ranges from 2% to 4% [53]. In contrast, near Disko Bay in western Greenland, glacial sources comprise up to 5% of the glacially modified water in the fjord [28].

The meltwater fraction observed in Andvord Bay within the 100-m surface layer is comparable to other estimates in WAP shelf waters. Low salinity surface waters (<33.5 PSU) are consistently observed off Anvers Island [58]. δ^18^O values are approximately -0.50‰ [52] resulting in a meteoric fraction of 2.5% to 3% meltwater in January.

Glacial meltwater is expected to contain sedimentary iron nanoparticles, which are ubiquitous in glacial ice [27]. The impact of these particles on water column turbidity in Andvord Bay and the concentration of suspended sediments were lower than those in Arctic fjords [23] where meltwater input, particularly associated with subglacial melting, can result in a substantial sediment loading. For instance, in Godthåbsfjord system situated in western Greenland, SPM concentration can reach >50 mg L^−1^ [59], while in Andvord Bay, maximum values did not exceed 10 mg L^−1^. While the optical signal from sedimentary particles in meltwater is relatively small in Andvord Bay, optical measurements such as particulate backscattering coefficient (*b*_*bp*_(442) and particulate beam attenuation coefficient (*c*_*p*_(660)) are sensitive enough to detect the presence of all sedimentary particles and facilitate the mapping of their distribution in the water column, and hence meltwater.

There is a discrepancy between spatial distribution of particulate backscattering coefficient and SPM measurements, where *b*_*bp*_(442) increases towards the glacier front and SPM does not (Fig 5a–5f). The difference between these two results suggests a large fraction of the glacial particles are smaller than the 0.7 μm nominal size of GF/F filters. This is consistent with the observation of the ubiquitous nature of iron nanoparticles in glacial meltwater [27].

Sea ice melting could also be contributing to the freshwater lens in Andvord Bay. However, sediment nanoparticles are known to be associated with glacial entrainment processes but not sea ice formation [60] [61]. In particular, in the surface and subsurface *b*_*bp*_(443) signal close to the glacial front (Fig 4, S3 Fig) suggests that the surface freshwater lens has similar origin. Sea ice melt also has a significantly higher δ^18^O signal when compared with that of glacial meltwater [53]. In Marguerite Bay, sea ice melt contributes ~0% meltwater during Austral summer, within the error (RMSD) for glacial meltwater fraction by δ^18^O (Table 2) and prediction by optical measurements (Table 4).

### Fjord phytoplankton

**Fig 10:**
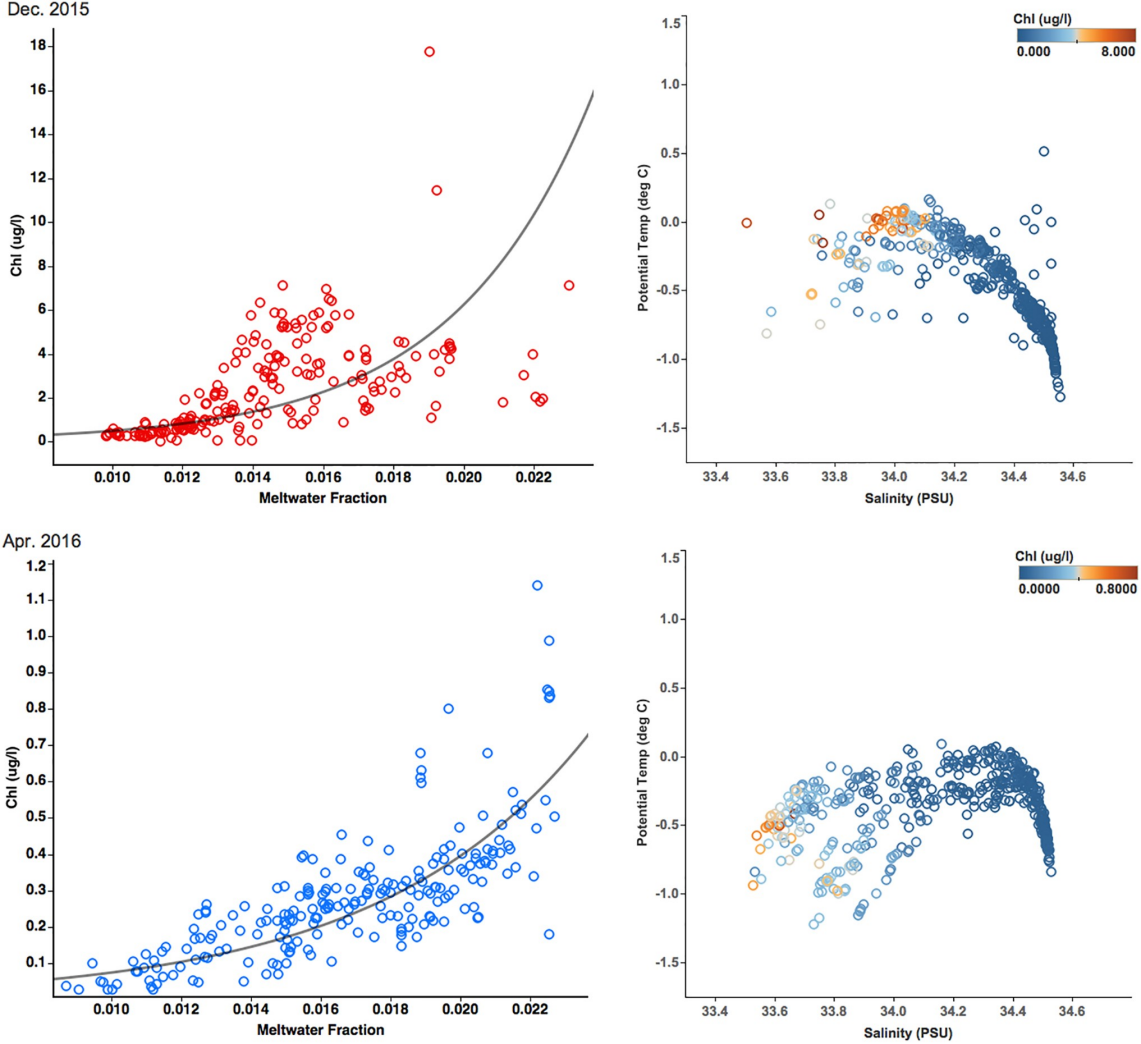
The relationship between meltwater fraction and chlorophyll-a concentration. Left panels: In the surface layer between 0 m and 40 m depth in Andvord Bay, the exponential regression between the two variables has a correlation coefficient of 0.66 and p-value of <0.0001 in December 2015, while the correlation coefficient is 0.82 and p-value is <0.0001 in April 2016. Right panels: T-S diagrams of Andvord Bay during December and April with color indicating chl-a concentration.

Meltwater in the fjord forms a surface layer (i.e., lens) where phytoplankton is retained, allowing for growth. Co-location of both variables, as seen in Figs 7, 3a and 3b, defines a relationship between phytoplankton abundance and meltwater fraction (Fig 10). An exponential regression between the two variables, established based on measurements above 40 m depth suggests a role of meltwater in facilitating phytoplankton growth (Fig 10). The correlation coefficient between the two variables is 0.66, p-value <0.0001 in December (Fig 10a) and 0.82, p-value <0.0001 in April (Fig 10b).

The influence of meltwater on phytoplankton abundance has also been found over the WAP shelf; between 1991 and 1999, chlorophyll concentration significantly increased with a decline in salinity attributed to glacial melt [8]. When freshwater input in a fjord does not contain significant sediment loading, it is expected to increasewater column stratification without significantly affecting underwater light field [62] [63]. While K_d_(PAR) significantly increased towards the glacial front in Andvord Bay, these values are incapable of hindering phytoplankton growth like the meltwater conditions in Greenland fjords (such as Godthåbsfjord in SW Greenland) [64]. In addition, meltwater can bring macro-nutrients and iron, both from glacial melt and/or upwelling of deep water, as observed in east Greenland fjords [62] [63] [65] (pers. comm. Forsch, K., Scripps Institution of Oceanography), resulting in well illuminated surface waters, rich in nutrients. These conditions are known to give rise to phytoplankton growth with small and fast-growing species like cryptohytes [66]. These clear, rich waters also sustain grazers resulting in an active marine food web [67]. In contrast, sub-Arctic Greenland fjords with high suspended sediment loading from meltwater, have high K_d_(PAR), are found to interfere with copepods’ feeding rates [59]. Thus, the low concentration of meltwater in Andvord Bay is sufficient to stabilize surface waters, without additional negative effects from high sediment loading [68] [69]. These waters are transparent, potentially enriched in nutrients from the glacial ice and nanoparticles, providing an ideal environment for phytoplankton growth [70].

Very low salinity is known to affect negatively phytoplankton growth. At Potter Cove (a fjord-like embayment on King George Island, northern Antarctic Peninsula), Hernando *et al*. [2015] experimented with two different salinity treatments: one with natural ambient salinity (34 PSU), and one with low salinity (30 PSU) [63]. They found that hypo-osmotic conditions favor water influx into the cells and cause an increase in turgor pressure and oxidative stress, resulting in instantaneous inhibition of phytoplankton growth rate and biomass accumulation, and reduced chl-a concentrations. Notably, these changes coincide with a gradual replacement of big centric diatoms by small pennate diatoms [63]. We did not observe this negative effect on phytoplankton in Andvord Bay (Fig 10). We interpret the salinities > 33.8 PSU are too high to affect phytoplankton or the dominant species were adapted to low salinity.

Phytoplankton abundance in Andvord Bay had a strong temporal variability, indicated by an order of magnitude decrease in chl-a concentration from December to April (Fig 3) This variability is likely due to the decrease in daylength from 18 h to 12 h and an increase in sun angle as the winter approaches [71]. Within the bay, the influence of meltwater on phytoplankton is likely expressed through a combined effect of water column stratification and nutrient addition induced by meltwater. In contrast, it is unlikely the presence of sedimentary nanoparticles significantly impacted underwater light field.

Chl-a and phaeo-pigment concentrations in Andvord Bay at z > 300 m are notable. Chl-a is the active pigment in algae, present only in live cells. Phaeo-pigments are products of chl-a degradation such that the combination of both variables represents carbon originating from algal biomass. Phytoplankton, and thus chl-a, are expected to concentrate in surface waters where PAR is available to support photosynthesis. Below the photic zone, phytoplankton decreases to negligible values, usually <1% of surface concentrations. In this way, the deep pigment concentrations are attributed to derived matter from phytoplankton production at the surface. This observation is consistent with motion picture data collected by a benthic camera system situated in the middle basin in 2015–2016. Footage revealed marine snow particles falling between Mid-January and early March (austral summer). The marine snow had a visually distinct dark green coloration, likely a result of sedimentation of a phytoplankton summer bloom between December and April (pers. comm. Smith, C.R., University of Hawai’i). The distribution of deep chl-a and phaeo-pigment concentrations in April also coincided with lower oxygen concentration at depth (Fig 2h), presumably indicating oxygen consumption by microbes during decomposition of the sinking organic particles.

### Fjord optical properties

#### Underwater light field

The underwater light field was largely influenced by phytoplankton in December, and by sedimentary particles in April. When particulate absorption and backscattering coefficients are compared with particulate beam attenuation coefficient, *c*_*p*_(660) is better correlated with *a*_*p*_(442) in December and with *b*_*bp*_(442) in autumn. Inorganic particles (such as glacial sediments) are generally more effective at scattering light than organic particles (such as phytoplankton) due to the sediments’ minerology and geometric shapes [72], while the presence of phytoplankton’s pigment make these organic particles more effective for light absorption [73]. In Andvord Bay, both types of particles existed concurrently but had different relative contribution to the particle assemblage in December and April. A clear *b*_*bp*_(442) signal near the glaciers was consistent through time suggesting the contribution from nanoparticles was constant. In contrast, *a*_*p*_(442), *a*_*NAP*_(442), and *a*_*ph*_(442) decreased in April, consistent with the one order of magnitude decrease in chl-a concentration (Fig 3).

The overall bio-optical profiles and their spatial distribution in Andvord Bay are comparable to those observed in northern Antarctic Peninsula and in Arctic fjords, with some distinct differences. Particulate backscattering and beam attenuation of light increase near the glaciers in Andvord Bay (Figs 4a–4d, 5a–5d). Mascarenhas *et al*. [74] also observed an increase in optical signal towards the head of fjords in central Norway, where they found an increase in *b*_*b*_(470) near the glacial fronts. This spatial pattern is also observed in Uummannaq Fjord and Vaigat-Disko Bay in western Greenland. These systems are strongly influenced by glacial meltwater runoff, which produces strong turbidity signals as a result of sediment plume discharge [30].

Particulate backscattering coefficients in Andvord Bay (0.001 m^-1^ to 0.015 m^-1^), are comparable to *b*_*bp*_(442) of 0.0004 m^-1^ to 0.0067 in northern Antarctic Peninsula [75] and are higher than *b*_*bp*_(488) over the shelf of 0.0015 m^-1^ to 0.006 m^-1^ [76] [77]. Our results in Antarctic fjords are in the range of values observed in the southeastern Beaufort Sea, as well as in the Chukchi Sea and western Beaufort [42]. In both the Arctic and Andvord Bay, *b*_*bp*_(λ) generally decrease with increasing wavelength for each sample. The high *b*_*bp*_(λ) values are associated with waters [75] near the glaciers in Andvord Bay, while the low values are found 170 km away, over the continental shelf adjacent to Anvers Island (S3 Fig). December *b*_*bp*_(λ) values have a larger range in comparison to those of April across all wavelengths. For comparison, *b*_*bp*_(λ) values in Beaufort Sea and Chukchi Sea varied six orders of magnitude [42]. The values ranged from the clearest offshore waters in the Beaufort sea where *b*_*bp*_(λ) was <0.005 m^-1^ to the highest *b*_*bp*_(λ) values, >0.1 m^-1^ from the Mackenzie River [42].

The difference between IOP values near and away from the glaciers in Andvord Bay is also reflected in AOP values. The largely absent spectral peaks in *R*_*rs*_(λ) between 360nm and 390nm in April 2016 indicate the significant shift from biotic to abiotic particles and CDOM absorption in comparison with December. The presence of nanoparticles also explains why while the range of *R*_*rs*_(λ) values in Andvord Bay are comparable to those found over the WAP shelf [77], the spectral shapes are different.

#### Sediments in meltwater

Near the glaciers, the shift in particle assemblage is attributed to the sedimentary particles from glacial meltwater (Figs 4a–4d and 5a–5d, S3 Fig). While surveys in Antarctic fjords have been scarce, limited studies from the past indicate the existence of “meltwater fingers” along the glacio-marine interface, including in Andvord Bay. Domack and Williams [1990] found a layered structure in the beam attenuation profile measured in this fjord, and speculated that these optical features are related to sediment loading as a result of glacial meltwater plumes [36]. In 2010, these persistent plumes near the glaciers were observed also in *c*_*p*_(660) profiles [78]. In this study, there was significantly higher *c*_*p*_(660) and *b*_*bp*_(442), in the inner basins near the glacier fronts (Table 3), with higher values found in April, confirming the persistence of these subsurface features. At Brialmont Cove in Hughes Bay, central Antarctic Peninsula, Domack and Williams (1990) observed quartz silt grains originating from basal, sediment-rich meltwater from the submerged glacier. Buoyant meltwater carries these particles to mid-water column, while turbulent mixing at depth also facilitates the transport and breaking of these floccules. These processes produce a midwater feature and turbidity at different depths near the glacier front and they can account for 87% of the total sediment load [79].

The absorption and backscattering of light by sediments is also attributed to their size distribution. At Brialmont Cove in Hughes Bay, 0.1–2 mm floccules were composed of individual grains between 5 and 50 μm [79] and captured by *c*_*p*_(660). Smaller grain sizes are often un-detected and therefore overlooked. It is likely that a substantial portion of the sediment particle assemblage are not effectively captured by the SPM measurement, which retains suspended sediment onto a GF/F filter that has a nominal pore size of 0.7 μm. This is consistent with our observations in Andvord Bay, where inorganic SPM did not exhibit a significant increase towards the glaciers in the surface layer (Fig 5e and 5f; Table 3), while several optical variables, expected to be affected by fine sediment load, presented significant response (Fig 5a–5d; Table 3). Bio-optical modeling indicates that particles <0.1 μm contribute significantly to light backscattering in the ocean [80]. Other studies have predicted also that 50% of particulate backscattering are due to particles < 0.2 μm [81]. These theoretical studies have demonstrated that realistic concentrations of sub-micrometer particles in the size range of 0.4–1 μm [82] can indeed produce significant backscattering signal [83]. In the context of Andvord Bay, suspended nanoparticles entrained in glacial meltwater in meltwater are expected to contribute to the *b*_*bp*_(442) and *c*_*p*_(660) signal.

### Optical modeling of meltwater fraction

δ^18^O measurement is a robust and accurate method for assessing meltwater fraction in the water column. This study shows that optical measurements can greatly complement conventional hydrographic tools to understand the spatial distribution of glacial meltwater (Figs 8 and 9, Table 4). More specifically, particulate backscattering and beam attenuation coefficients were sensitive to the low meltwater concentrations observed in Andvord Bay. The predicted meltwater fraction based on optical methods demonstrated good agreements with *in-situ* estimates based on δ^18^O values (Figs 8 and 9), and the observed and predicted meltwater fractions in this study are comparable to the range of values previously found in this region [52] [53].

The predicted meltwater fraction based on specific IOPs (Q_i,iop_), *b*_*bp*_(442), *a*_*NAP*_(442), and temperature, allow Model 1 to encompass both backscattering and absorption of inorganic sediment particles from glacial meltwater. *b*_*bp*_(λ) is expected to be associated with the sediment loading [80] [84]. Concurrently, the presence of small particles (<0.7 um) is likely linked to large particles retained by the GF/F filter (>0.7 um), hence a_*NAP*_(442) in Model 1 contributed to the overall statistical significance. Despite this, GF/F-based measurements of large particles alone cannot predict the concentration of small particle assemblage (Table 3). This leads to the speculation that the small particles are disproportionately linked to large particle concentrations (eg. as an exponential function), thus higher concentrations of small particles can be masked by the presence of large particles.

Modeling with reflectance ratio of suspended sediments modified from Tassan (1994) [56] and Sravanthi *et al*. [55] provided a significant prediction of meltwater fraction and distribution in the water column (Model 2, Table 4, Fig 9). This further confirms that the AOP variables are responding to the IOP sensitivity to the sediment loading in meltwater (See Fig 5a and 5b). Same as the algorithm by Sravanthi *et al*., the model needed further adaptation to local conditions, attributed to a difference in Antarctic water properties with respect to the global ocean average [56] or the Indian Ocean [55], where the algorithms (Eqs 13 and 14) were developed and applied. While Sravanthi *et al*. [55] added 2 coefficients to the original formulation predicting sediment loading based on one cumulative AOP variable, creating a non-zero intercept (α) and adjusting the slope (β), our adaptation of the method created variables in a multivariate linear model, ${\alpha_{4}(R(555)+R(625))+\alpha_{5}(\frac{R(625)}{R(490)})}^{2}$, that produced statistical significance for our study region with high correlation coefficients (r = 0.81 and 0.84 in December and April, respectively, Table 4). The first term is sensitive to sediment loading at the wavelengths of low phytoplankton and CDOM absorption. In Andvord Bay, *R*_*rs*_(λ) shows a maximum between 475 nm and 555 nm, and a minimum around 625 nm (Fig 6). The band ratio represents the correction by phytoplankton absorption at the wavelength of maximum steepness, similar to results derived from algorithms developed for the Indian Ocean and global ocean average.

There are certain limitations to this approach of estimating glacial meltwater fraction based on optical measurements. First, the determination of meltwater by δ^18^O based on the two end-member mixing assumption does not account for sources from sea ice melt (see Discussion section a). As mentioned before, sea ice melt could have had a small signal in December, but did not seem present in April. In addition, this approach does not differentiate between meltwater from glaciers and from bergy bits as both originate from glacial ice and have similar δ^18^O signal [50] [52] [85] [86].

The multivariate linear regression approach has certain assumptions which require further diagnostics for verification. The standard linear model assumptions are linearity of the relationship, normality of residual errors, homogeneity of residuals’ variance, and presence of influential values. The linear relationships between optical variables and meltwater fraction are indicated by their high r values (Table 4); there are also no outstanding outliers to significantly influence the models (Figs 8 and 9). The QQ plot of residuals were used to check the normality assumption. The residuals follow the reference line (S4a, S4b, S4e and S4f Fig) indicating normal distribution. Homoscedasticity is verified by the scale-location plots to check the homogeneity of the residuals’ variance. The predicted values spread along a horizontal line indicating homoscedasticity of our data used for developing Model 1 and Model 2 (S4c, S4d, S4g and S4h Fig). In this way, the error of the statistics applied in modeling are minimal and the models are robust (Table 4).

## Conclusions

Glacial meltwater input in fjords and other coastal Antarctic regions has been associated with the regional warming [9]. The meltwater discharge can impact surface stratification and increase nearshore turbidity which influences underwater light field as shown in this study. Meltwater can also have secondary effects on Antarctic coastal ecosystems by influencing the timing of sea ice formation and promoting phytoplankton growth [7]. In Andvord Bay, we observed a relatively weak meltwater process near the glacio-marine interface with Bagshawe glacier. Concurrent *in-situ* optical measurements, especially particulate backscattering coefficient and particulate beam attenuation coefficient, were found to detect the presence of fine sediment loading. The presence of sedimentary nanoparticles, in combination with more negative δ^18^O and lower salinity, were attributed to the presence of meltwater. These nanoparticles were likely missed by sampling with standard GF/F (0.7 μm) filters. These optical features were utilized to model the spatial distribution of meltwater fraction in Andvord Bay (Model 1). Model 2 better correlates with *in-situ* meltwater measurements than Model 1 due to the integrative effect of the AOP variables. The models developed in this study can potentially be applied to remote sensing datasets to detect meltwater presence at the sea surface. In addition to the optical properties of glacial meltwater, we also documented a significant temporal variability in phytoplankton concentration, where surface chl-a decreased one-order of magnitude from December to April. Phytoplankton sedimentation was observed as deep chl-a and phaeo-pigments (z > 300m), increasing towards the end of the austral growing season.

## Supporting information

S1 Fig Temperature-salinity diagram and “Gade Line” between oxygen isotope ratio and salinity. T-S Diagram of cruise data in (**a**) December 2015 and (**b**) April 2016, and (**c, d**) δ^18^O values along salinity gradient from each season. Each data point is a discrete water column sample, and the color of the point indicates its sampling station’s distance from the glaciers, which are situated in the inner basins at the head of Andvord Bay.
(TIF)

S2 Fig Cross section plots of particulate absorption, non-algal absorption, and CDOM absorption coefficients at 442 nm. (**a, b**) Particulate absorption coefficient at 442 nm. (**c,d**) Non-algal particle absorption coefficient at 442 nm. (**e, f**) CDOM absorption coefficient at 442 nm.
(TIF)

S3 Fig Profiles of particulate backscattering coefficient at 442 nm. December 2015 (above) and April 2016 (below), where high backscattering signals are observed in the inner basins (blue) in comparison to the significantly lower values found on the shelf (red); the profiles in black are the rest of the sampling stations between inner fjord and the shelf.
(TIF)

S4 Fig Multivariate linear regression model assumption diagnostics. Diagnostic plots for Models 1 and 2 which results are presented in Figs 8 and 9. Normality of residuals presented in normal Q-Q plots (upper panels) and homogeneity of residuals variance presented in location-scale plots (lower panels).
(TIF)
