# Supplementary figures and images for: The optical and biological properties of glacial meltwater in an Antarctic fjord
Source: PLoS One. 2019 Feb 6;14(2):e0211107. doi: 10.1371/journal.pone.0211107 (PMC6364926; doi:10.1371/journal.pone.0211107)

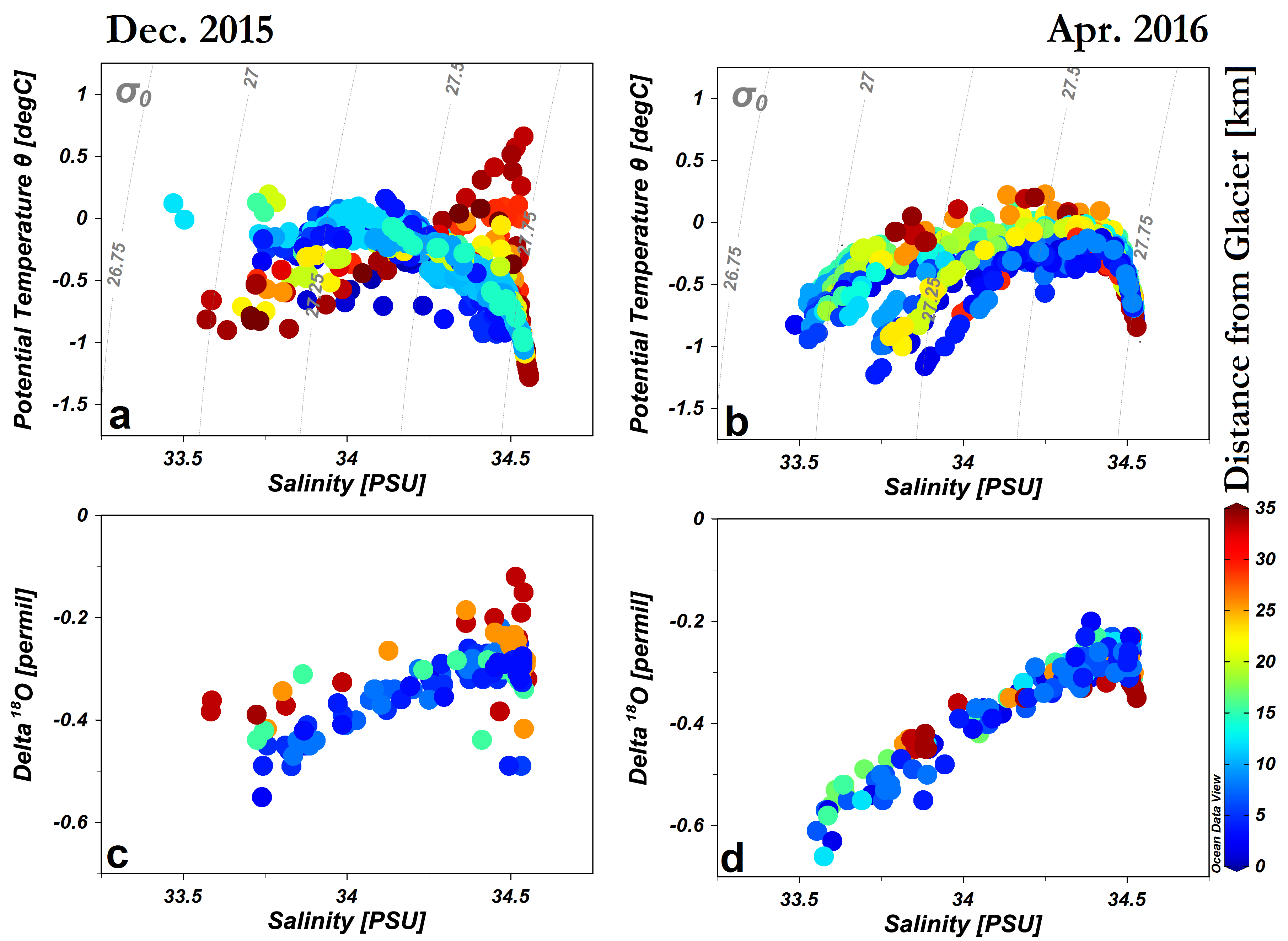

Supplement: S1 Fig — T-S Diagram of cruise data in (a) December 2015 and (b) April 2016, and (c, d) δ18O values along salinity gradient from each season. Each data point is a discrete water column sample, and the color of the point indicates its sampling station’s distance from the glaciers, which are situated in the inner basins at the head of Andvord Bay. (TIF) [file pone.0211107.s001.tif]

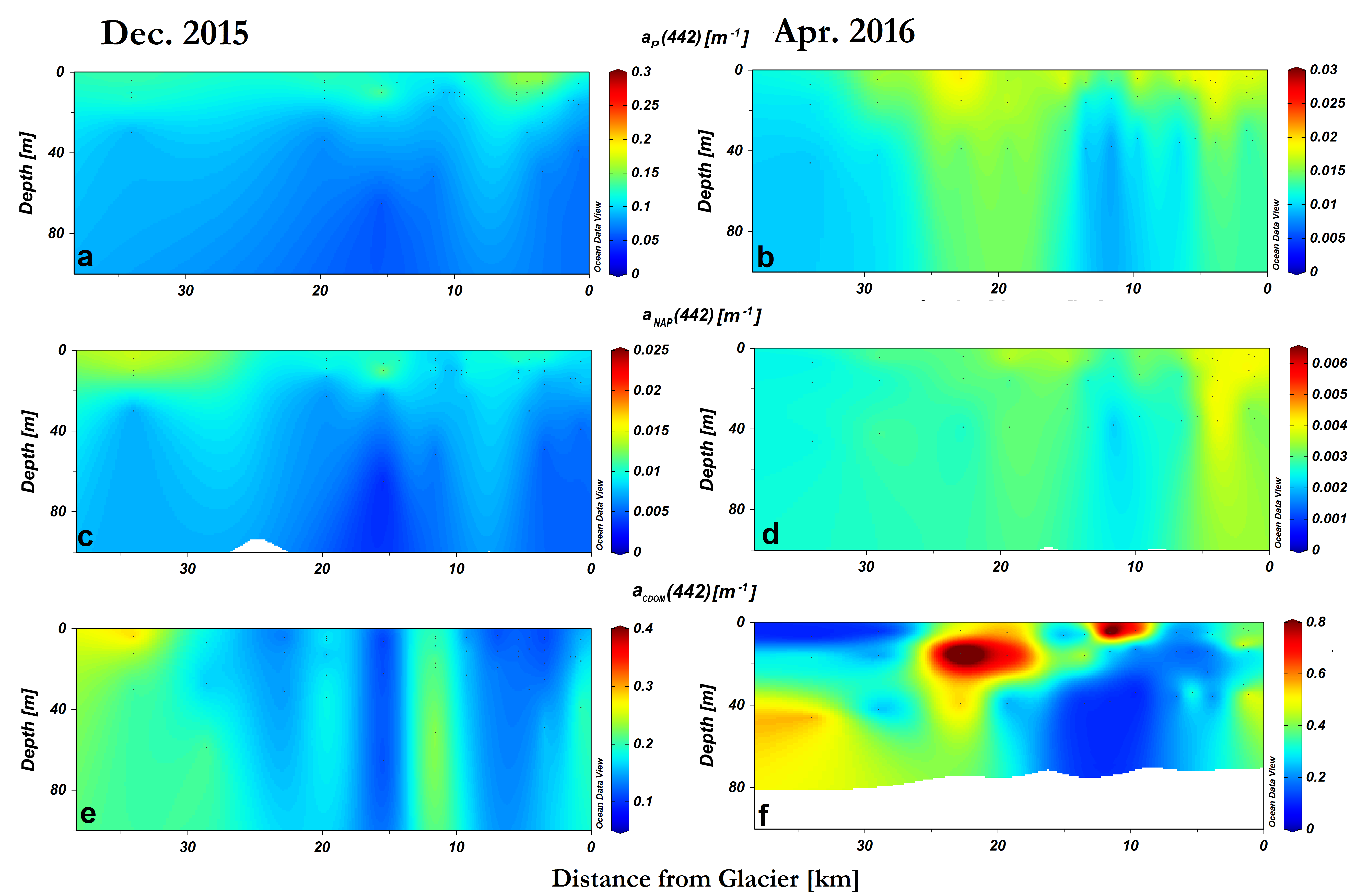

Supplement: S2 Fig — (a, b) Particulate absorption coefficient at 442 nm. (c,d) Non-algal particle absorption coefficient at 442 nm. (e, f) CDOM absorption coefficient at 442 nm. (TIF) [file pone.0211107.s002.tif]

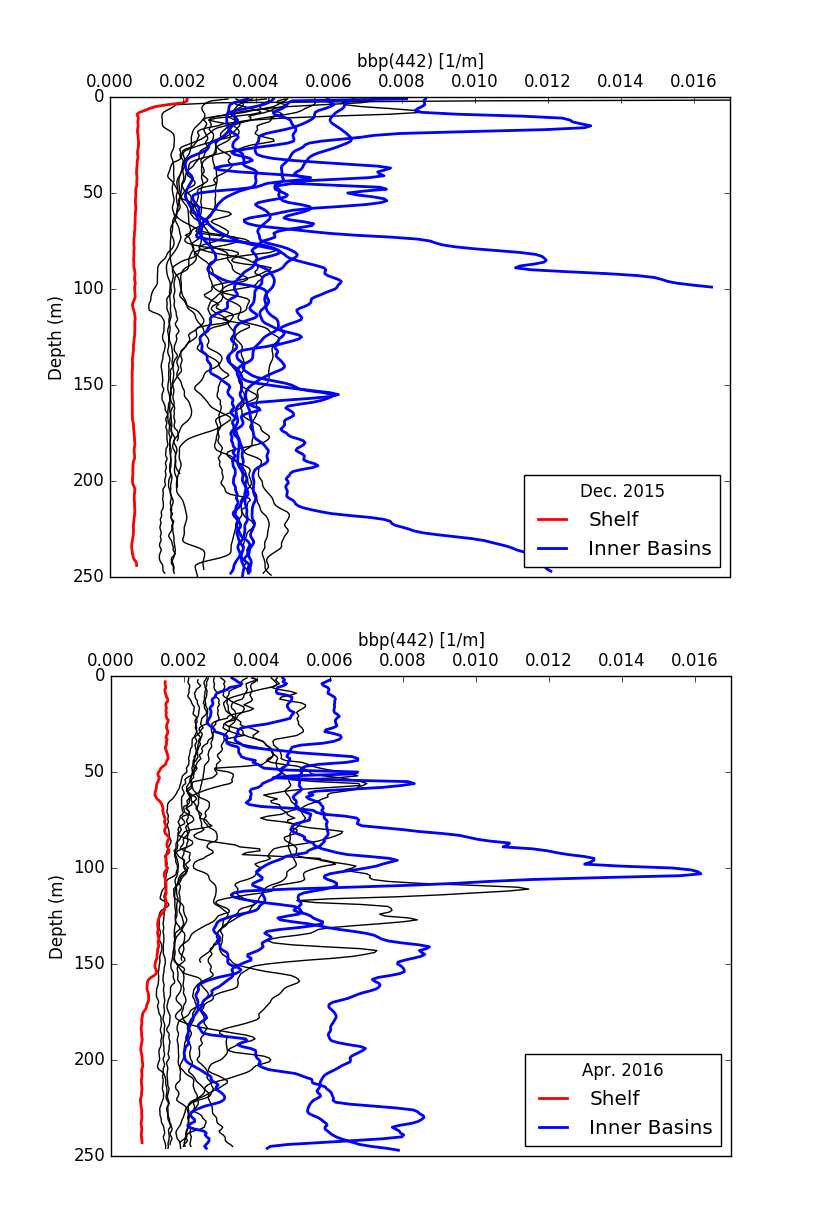

Supplement: S3 Fig — December 2015 (above) and April 2016 (below), where high backscattering signals are observed in the inner basins (blue) in comparison to the significantly lower values found on the shelf (red); the profiles in black are the rest of the sampling stations between inner fjord and the shelf. (TIF) [file pone.0211107.s003.tif]

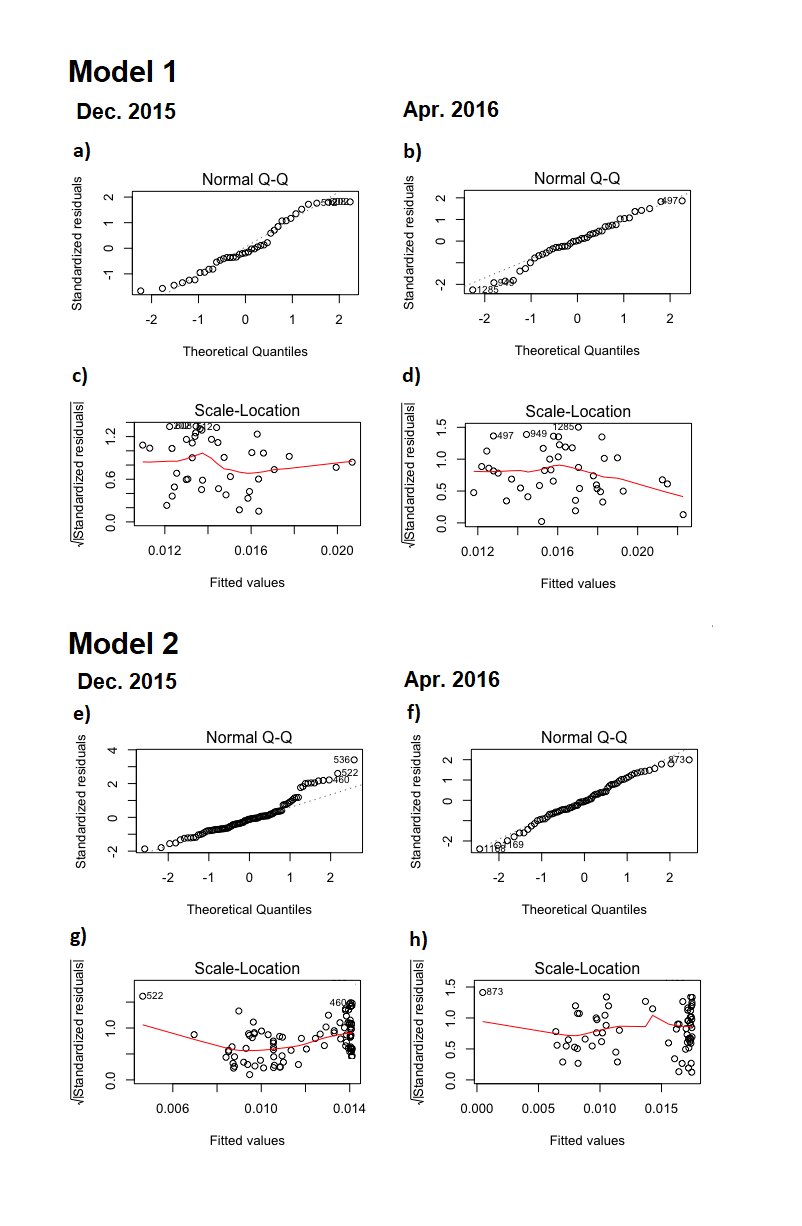

Supplement: S4 Fig — Diagnostic plots for Models 1 and 2 which results are presented in Figs 8 and 9. Normality of residuals presented in normal Q-Q plots (upper panels) and homogeneity of residuals variance presented in location-scale plots (lower panels). (TIF) [file pone.0211107.s004.tif]
